# Bipartite life cycle of coral reef fishes promotes increasing shape disparity of the head skeleton during ontogeny: an example from damselfishes (Pomacentridae)

**DOI:** 10.1186/1471-2148-11-82

**Published:** 2011-03-30

**Authors:** Bruno Frédérich, Pierre Vandewalle

**Affiliations:** 1 Laboratoire de Morphologie Fonctionnelle et Evolutive, Institut de Chimie (B6c), Université de Liège, B-4000 Liège, Belgium

## Abstract

**Background:**

Quantitative studies of the variation of disparity during ontogeny exhibited by the radiation of coral reef fishes are lacking. Such studies dealing with the variation of disparity, i.e. the diversity of organic form, over ontogeny could be a first step in detecting evolutionary mechanisms in these fishes. The damselfishes (Pomacentridae) have a bipartite life-cycle, as do the majority of demersal coral reef fishes. During their pelagic dispersion phase, all larvae feed on planktonic prey. On the other hand, juveniles and adults associated with the coral reef environment show a higher diversity of diets. Using geometric morphometrics, we study the ontogenetic dynamic of shape disparity of different head skeletal units (neurocranium, suspensorium and opercle, mandible and premaxilla) in this fish family. We expected that larvae of different species might be relatively similar in shapes. Alternatively, specialization may become notable even in the juvenile and adult phase.

**Results:**

The disparity levels increase significantly throughout ontogeny for each skeletal unit. At settlement, all larval shapes are already species-specific. Damselfishes show high levels of ontogenetic allometry during their post-settlement growth. The divergence of allometric patterns largely explains the changes in patterns and levels of shape disparity over ontogeny. The rate of shape change and the length of ontogenetic trajectories seem to be less variable among species. We also show that the high levels of shape disparity at the adult stage are correlated to a higher level of ecological and functional diversity in this stage.

**Conclusion:**

Diversification throughout ontogeny of damselfishes results from the interaction among several developmental novelties enhancing disparity. The bipartite life-cycle of damselfishes exemplifies a case where the variation of environmental factors, i.e. the transition from the more homogeneous oceanic environment to the coral reef offering a wide range of feeding habits, promotes increasing shape disparity of the head skeleton over the ontogeny of fishes.

## Background

A primary aim of evolutionary biology is to explain the origin, structure and temporal patterns of phenotypic diversity. Many studies have proposed adaptive explanation for phenotypic variation and have focused on the role of selection in shaping patterns of diversification. Divergent selection is expected to enhance adaptive differences through time (evolutionary and ontogenetic scales). Selection may occur at any life stage and be specific to one life stage, leading to the potential for stage-specific adaptation [1]. Despite its obvious importance, very little is known about the role of ontogeny in adaptive divergence and rigorous studies focusing on the variation of phenotypic diversity over ontogeny in various zoological groups are needed.

Coral reef fishes represent one of the most diverse assemblages of vertebrates, and moreover studies dealing with their morphological diversity are numerous [2-10]. These studies have mainly focused on the adult stages and fewer have addressed ecomorphological variation through the ontogeny of reef fishes [e.g. [11,12]]. The majority of coral reef fishes have a complex life-cycle with two distinct phases: (1) a dispersive pelagic larval phase and (2) a sedentary demersal adult phase associated with the coral reef environment. The larval phase ends at reef settlement [13]. In contrast to reefs, the open water environment is thought be more homogenous, especially with regard to the diversity of habitats. Conversely, intrinsic factors of the coral reefs such as high productivity, high spatial and ecological complexity, and high trophic diversity may be involved in promoting the high standing levels of fish diversity [14]. It is thus surprising that quantitative studies on the ontogeny of the radiation of coral reef fishes are lacking. Such studies dealing with the variation of disparity, i.e. the diversity of organic form [15], over the ontogeny could be a first step in detection of evolutionary mechanisms of diversification in these fishes. By studying their ontogeny, via changes in shape with size (i.e. allometry), it is possible to gain a clearer understanding of the timing of selective pressures in coral reef fishes [1].

To our knowledge, very little quantitative data exists on adaptive allometry and on the ontogeny of shape diversification within zoological groups in general and fish clades especially [but see [1,16,17]]. Zelditch et al. [18] have focused on the dynamic of body shape disparity over ontogeny in piranhas and highlighted that the disparity decreases significantly and substantially over ontogeny within this particular clade. Adams and Nistri [19] investigated ontogenetic trajectories of foot morphology in eight species of European plethodontid cave salamander and showed the disparity of adult foot morphology was significantly lower than in juveniles. Two broad categories of factors have been suggested for explaining these ontogenetic patterns of disparity [20]: the external or "ecological" constraints such as the availability of ecological space [21,22], and the internal constraints such as developmental, genetic or functional constraints [18,23,24]. New studies in various taxa selected according to their life-cycle and their ecological diversity are needed. They should give further insights into the roles of external and internal constraints shaping the levels and patterns of disparity over ontogeny.

Including more than 300 species living in coral reef environments, the damselfishes (Pomacentridae) represent one of the most successful radiations of coral reef fishes [3,14,25,26]. Similar to the great majority of coral reef fishes, they have a bipartite life-cycle. In the pelagic environment, all damselfishes larvae feed on planktonic copepods [27]. At the adult stage, three trophic guilds are commonly recognized [25,28]: the pelagic feeders sucking planktonic copepods; the benthic feeders grazing filamentous algae or biting coral polyps; and an intermediate group feeding on planktonic prey, small benthic invertebrates and algae in variable proportions. Consequently, we can consider that damselfishes have a higher trophic diversity at their adult stage, and thus we can expect that functional demands lead to an increase in morphological disparity over ontogeny. Indeed, while all species might be expected to be adapted to catch small planktonic prey during the pelagic larval phase, adult damselfishes would be expected to have specific morphological adaptations allowing optimal prey catching and processing. For example, planktivorous and algivorous species have muscles and skeletal shapes optimizing suction feeding and grazing, respectively [3,4,29].

The evolution of damselfishes exemplifies a case of reticulate adaptive radiation [3] in which morphological divergence at speciation has been associated with the repeated convergence on a limited number of ecotypes: algivory, omnivory and planktivory. Among the Pomacentridae, algivory and omnivory have both arisen seven times, planktivory four times, and feeding on scleractinian coral polyps twice [3,25,26].

The study of ontogenetic allometry can reveal differences in developmental patterns among species underlying their morphological differentiation. How differences in developmental patterns generate species divergence has been successfully addressed in many groups, including trilobites [e.g. [30]], fishes [e.g. [17]], newts [e.g. [31]], reptiles [e.g. [32]], rodents [e.g. [33]], primates [e.g. [34]] and humans [e.g. [35]]. The pattern of ontogenetic shape changes can be described by the allometric trajectory of an organism plus the rate at which it proceeds along the trajectory [36]. Morphological divergence among taxa sharing a common allometric trajectory could be due to changes in the rate or the duration of development (i.e. rate and event heterochrony [37]). Divergence could result from the directional change in the allometric trajectories (i.e. allometric repatterning [37]), coupled or not to alterations in the rate and/or the timing of development. In the present study, we explore whether and how differences in allometric trajectories can shape the ontogenetic dynamic of morphological disparity in damselfishes.

We used geometric morphometrics to examine the patterns of morphological diversification among damselfishes throughout their ontogeny. Four functional units of the head skeleton were separately studied: the neurocranium, the unit «suspensorium and opercle», the mandible and the premaxilla. All these skeletal units are movable elements involved in prey catching [38]. The aims of the present study were to: (1) test the hypothesis that larval morphologies are more similar (less disparate) than the morphologies of juveniles and adults, (2) compare the patterns of shape disparity, i.e. the distribution of shapes and the dimensions along which shapes are most disparate, at different ontogenetic stages, (3) compare ontogenetic trajectories to identify the evolutionary changes in developmental parameters (i.e. allometric patterns, rate of shape changes, amount of shape changes undergone over the course of ontogeny) shaping the level and the pattern of morphological disparity, and (4) explore the relationships between the developmental parameters, phylogenetic data and ecological data.

## Methods

### Sample and data collection

**Figure 1:**
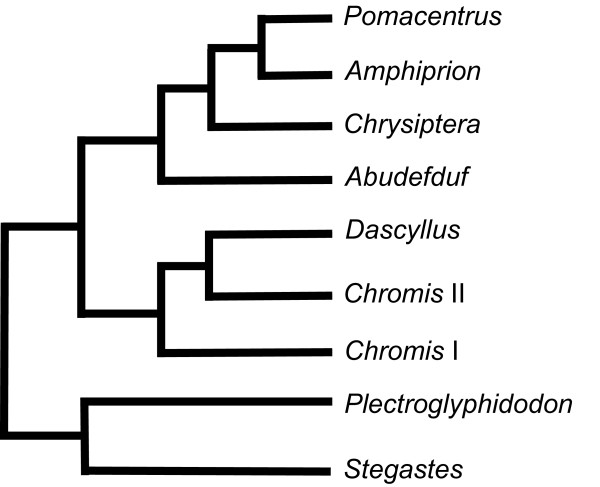
**Phylogenetic relationships among the most specious genera of Pomacentridae**. The tree topology drawn according to molecular phylogenetic studies [26] is simplified because all genera are not shown.

Our study species belong to 6 of the most specious genera of Pomacentridae [25], the phylogenetic relationships of which are illustrated on Figure 1. They represent every trophic group known at the adult stage [25,28]: zooplanktivorous species (*Abudefduf sexfasciatus*, *Chromis *sp and *Dascyllus trimaculatus*), algivorous species (*Stegastes nigricans*, *Chrysiptera brownriggii *and *Chrysiptera glauca*) and species belonging to the intermediate group (*Dascyllus aruanus *and *Pomacentrus pavo*). Within the intermediate group, *D. aruanus *is a mainly carnivorous species feeding on planktonic and benthic copepods [39] when *P. pavo *feeds on zooplankton and filamentous algae [25]. A total number of 406 specimens of damselfishes were analyzed (Table 1). The sample of each species represents a complete ontogenetic series from larval (i.e. larvae settling reef) to adult specimens. The samples of two *Chromis *species, *Chromis viridis *(*n *= 15) and *Chromis atripectoralis *(*n *= 21), were pooled to build a single ontogeny referred as *Chromis *sp in this study. These two very close species are named the blue green damselfishes, differing only by the coloration of the pectoral fin base [40]. All juvenile and adult specimens were collected by the authors in the lagoon or on the outer reef slope at Toliara (Mozambique Channel, Madagascar) in June 2004, October 2006 and November 2007; at Moorea Island (Society Archipelago, French Polynesia) in June 2007; and at Rangiroa atoll (Tuamotu Archipelago, French Polynesia) in July 2007 after being anaesthetized by a solution of quinaldine. All larvae were collected by the authors at Moorea Island and Rangiroa atoll. Larvae were caught during settlement events with a crest net (1.5 m wide × 0.75 m height × 5 m length, 1 mm mesh net) fixed to the substratum similar to one used by [41]. At Moorea Island, crest nets were placed on the reef crest. At Rangiroa atoll, the crest net was positioned in a hoa or channel through which water entered the lagoon. Fish larvae captured in crest nets during the night were collected at dawn. Fishes were preserved in neutralised and buffered 10% formalin for ten days and then transferred to 70% alcohol. All specimens were cleared and stained with alizarin red S [42] in order to display the osseous skeleton.

**Table 1:** Studied species

*Species*	*Abbreviations*	*PLD (days)*	*SL^settlement^*	*SL^max^*	*Size range (SL, mm)*	*n_neuro_*	*n_susp_*	*n_mand_*	*n_premax_*
*Abudefduf sexfasciatus*	*A. sex*	16-18	10-11	140	10-102	41	38	43	41
*Chromis *sp	*Chromis*	18-29	6-8	70	7-77	33	36	35	34
*Chrysiptera brownriggii*	*C. bro*	18-29	14-15	60	14-55	50	52	55	55
*Chrysiptera glauca*	*C. gla*	15-24	13-16	80	13-68	37	39	36	37
*Dascyllus aruanus*	*D. aru*	16-26	7	65	7-60	55	55	58	59
*Dascyllus trimaculatus*	*D. tri*	21-30	10-11	110	10-100	65	67	69	62
*Pomacentrus pavo*	*P. pav*	20-27	13-16	85	13-63	66	74	68	74
*Stegastes nigricans*	*S. nig*	24-34	14-15	115	14-127	37	37	37	36

PLD, pelagic larval duration [73]; SL, standard length; SL^settlement^, standard length at settlement [74]; SL^max^, maximum standard length [25] in millimeter; *n*, number of specimens for each structure.

**Figure 2:**
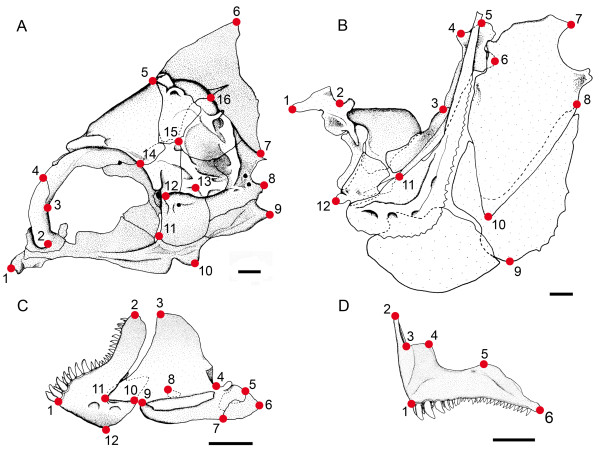
**The morphologically homologous landmarks used in the analysis of the ontogeny of damselfish skull shape**. (A) Neurocranium, (B) «suspensorium and opercle» unit, (C) mandible and (D) premaxilla of *Dascyllus aruanus *illustrating the various landmarks. Refer to [4] for the description of each landmark. Scale bars = 1 mm.

One of us (B.F.) dissected fish heads and collected two-dimensional anatomical landmarks (LMs) on lateral views of the neurocranium, the unit «suspensorium and opercle», the mandible and the premaxilla using a Leica M10 binocular microscope coupled to a camera lucida. Lucida images drawn on sheets were then scanned and the x, y coordinates of LMs were digitized using TpsDig (version 1.40). The configuration of LMs of each skeletal unit used for the analysis is shown on Figure 2. Sixteen homologous LMs were defined on the neurocranium, 12 on the unit «suspensorium and opercle», 12 on the mandible and 6 on the premaxilla, forming four separate data sets. Landmarks are described in [4] despite the fact that the number of LMs used for defining the unit «suspensorium and opercle» is lower than in our previous study of adult damselfishes [4] because some LMs could not be easily and precisely discriminated in larvae. The size of samples differed slightly between structures because some units were damaged before or during the dissection (Table 1).

### Geometric morphometrics

We used Procrustes-based geometric morphometrics to study shape variation and dynamics of shape disparity throughout ontogeny [43-46]. For each skeletal unit, the digitized landmark configurations were subjected to a Generalized Procrustes Analysis (GPA) in order to remove non-shape variation (location, orientation and scale) [47,48]. The "grand mean", i.e. the consensus shape of all specimens and shape variables were then generated as partial warp scores (PWs) including both uniform and non-uniform components [44,49]. The centroid size (CS) of the structure was also computed as the square root of the sum of the squares of the distances from all LMs to their centroid [44]. Allometry refers to the pattern of covariation between measures of size and shape [36]. Age information is not available for our specimens, so we use size as ontogenetic scale (i.e. ontogenetic allometry).

#### 1. Comparing models of ontogenetic allometries

The allometric patterns of shape variation were analyzed using linear multivariate regression of PWs on log-transformed size (ln-CS) [48,50-52]. The null hypothesis that shape develops isometrically was tested in all species using TpsRegr (Version 1.34). The fit of the regression models was evaluated by the explained variance of the model and by a permutation test based on a Generalized Goodall's F-Test with 10,000 permutations.

Differences in allometric models among species were tested by a MANCOVA, testing the null hypothesis of homogeneity of linear allometric models. In these tests, shape variables (PWs) are considered as dependent variables, size (ln-CS) as covariate and species is grouping factor. As suggested by Zelditch et al. [53] and Webster and Zelditch [37], two factors can explain differences in allometric models: (a) the divergence of allometric trajectories and (b) the rate of shape changes. Consequently, we estimated and compared both factors plus two other parameters: shape at settlement (i.e. the starting point of the ontogenetic trajectories) and the length of the ontogenetic trajectory, which is a function of the rate and duration of development. As these parameters are estimated by linear multivariate regressions, they assume a linear relationship between shape and ln-CS. Plots of Procrustes distance from the mean larval shape (see below), and the variance explained by the models are used to assess the validity of log-linear models (Goodall's F-test). This is reasonable for our data because the regression of shape on ln-CS explains a large proportion (up to 88%) of shape variance for each structure in every species (see Additional File 1).

#### 1.a. Comparing larval shapes at settlement (starting point of ontogenetic trajectories)

The samples of settling larvae are limited (≤ 10 specimens). Consequently, we used a standardized regression residual analysis to estimate and compare larval shapes at settlement [18,54,55]. From the multivariate regression of shape on ln-CS, the non-allometric residual fraction is standardized by Standard6 (IMP-software). «Standardized» data sets of larvae with their respective SL (see SL_settlement _in Table 1), which are the predicted shapes of the entire population at these sizes, are generated. Multivariate analyses of variance (MANOVA) were performed using partial warp scores, followed by *a posteriori *tests of pairwise comparisons to test shape differences among species. The statistical significance of the pairwise differences was tested by a resampling-based *F*-test and the results of CVA assignment tests were also examined. MANOVAs were performed using Statistica 8.1 (Statsoft 2007). Pairwise *F*-tests were done in TwoGroup6 (IMP-software) and misclassification rates were given by CVAGen (IMP-software).

#### 1.b. Comparing ontogenetic trajectories in shape space

The differences in trajectories of shape changes were analyzed by comparing the angle between the species-specific multivariate regression vectors using VecCompare6 (IMP-software). This test is described in detail in [48,53] and was already exemplified in a previous study of allometry in damselfishes [50]. Here, a within-species vector is composed of all regression coefficients of the shape variables (PWs) on the log-transformed CS. The range of angles between such vectors within each species is calculated using a bootstrapping procedure (N = 400). This range was then compared with the angle between the vectors of both species. If the between-species angle exceeds the 95% range of the bootstrapped within-species angles, the between-species angle is considered significantly different, and thus the allometric trajectories are different. Angles were computed pairwise between allometric vectors, and the resulting interspecific dissimilarity relationships (angles) between the allometric trajectories were summarized with scatter plots calculated using nonmetric multidimensional scaling (NMDS). Differences between a reference shape (i.e. larval shape at settlement) and a target shape (i.e. adult shape) can be illustrated with deformation grids (interpolating function "thin plate spline" or TPS [44]). Multivariate regression models of shape on size for each unit were used to show graphical illustrations of ontogenetic allometries in each species. Deformation grids illustrating these ontogenetic allometries were obtained from TpsRegr (Version 1.34).

#### 1.c. Comparing rates of shape changes during ontogeny

The dynamics of shape change (developmental rate), defined as the rate of shape changes per unit of size in this study, was estimated for each species using the Procrustes distance (PD), the metric defining shape dissimilarity in the Kendall shape space [45]. PD between each specimen and the average larvae were regressed on ln-CS in all species separately. The rate of divergence away from the average larval shape was compared among species using the slope of the regressions with Regress6 (IMP-software) ([for detailed explanations on this methodology, see [48,56]). Because the relationships between PD and ln-CS are close to linear (see Additional File 2), these can be statistically compared by ANCOVA.

#### 1.d. Comparing lengths of ontogenetic trajectories

The length of the ontogenetic trajectories is used to compare the net amount of shape change undergone over post-settlement ontogeny in the eight species. These lengths were calculated as the PD between the average larval shape at settlement and at the maximum adult body size. Confidence intervals are placed on these lengths by a bootstrapping procedure detailed in [18]. Because our analyses are based on size-standardized data, so the bootstrapping procedure also takes the uncertainties of the regression into account. The calculations were done using DisparityBox (IMP-software).

We focused on the relationships between the studied developmental parameters, phylogenetic data and the diet of each species. Moreover, correlation analyses (Pearson r) were used to test the relationships between some developmental parameters, i.e. the dynamic of shape changes or the length of ontogenetic trajectories, and other species-specific characters, i.e. the pelagic larval duration (PLD) and the size variation undergone during post-settlement growth.

#### 2. Measuring and analyzing shape disparity

We used the same methodology as [18] to measure morphological disparity (MD) at three ontogenetic stages: (1) at the mean size observed at settlement (Table 1), (2) at a common size of 60 mm SL for every species and (3) at the species-specific maximum adult body size (MAX SL). The intermediate size (60 mm SL; at such a size, some species are already adults and others are always at the juvenile stage) was arbitrarily chosen to estimate the MD when fishes are already settled on coral reef and when size difference among species are eliminated. For these analyses, we used "standardized" data sets, which are the predicted shapes of the entire population (see Table 1) at these stages.

MD was calculated with the following equation:

where *d_j _*= PD between the mean shape of species j and the grand mean shape (i.e. consensus shape). *N *= total number of species. The level of disparity among the eight studied species was calculated for each skeletal unit (i.e. disparity at the family level). Then, we calculated the disparity level at the three studied ontogenetic stages within each trophic group. As only two species of the intermediate group were studied (i.e. *D. aruanus *and *P. pavo*), we could not calculate the disparity level of this trophic group. Consequently, both species were related to the two other trophic groups. As *P. pavo *commonly grazes algae, it was grouped with algivorous species. Conversely, *D. aruanus *was groupd with the zooplanktivrous species. All the calculations were done by DisparityBox (IMP-software), which also uses a bootstrapping procedure to place confidence intervals on this measure. We refer to [18,48] for a detailed explanation of this methodology.

In addition to measuring the level of shape disparity, we also examined its pattern of variation, i.e. the distribution of shapes and the dimensions along which shapes are most disparate. This exploration is informative about the dynamic nature of disparity. For example, larvae and adults can have the same level of disparity but they can occupy two different sub-spaces (hyperplanes, i.e. "flat" surfaces of more than two dimensions embedded in higher dimensional space) of the shape space. Biologically, this would mean that the patterns of variation have been re-organized or re-structured to lie along very different pathways. The structures of disparity in different ontogenetic stages can be compared by comparing variance-covariance matrices. First, visual exploration of shape variations in the sub-space defined by the first two relative warps (Relative Warps analysis is equivalent to a Principal Components Analysis [PCA] of shape variables when the scaling factor α = 0, [49]) allow to check if the three ontogenetic stages (i.e. settling larvae, 60 mm SL, MAX SL) share the same hyperplane. Then, we used the program SpaceAngle (IMP-software) to compute the angle between hyperplanes and determine if the between-ontogenetic stages angle is no larger than the within-ontogenetic stages range. This program allows to specifically test if every ontogenetic stage occupied the same subspaces of the morphospace. SpaceAngle (IMP-software) used a method developed by [57] based on PCA. See [48] for detailed explanations on this approach but the angle between two subspaces embedded in a common higher dimensional space can be defined as the angle through which one subspace must be rotated to match the other. If the angle between two subspaces (i.e. between-ontogenetic stages angle) does not significantly differ from zero, we may not reject the null hypothesis (H_0_) in which specimens of two ontogenetic stages occupy the same subspace. The significance of the angle was determined by a bootstrapping procedure (N = 400) similar to that applied for comparisons of allometric vectors (see above). Relative warps analyses of standardized shape data at the three ontogenetic stages were performed using PCAGen (IMP-software) and the angles between subspaces defined by the first two PCs were calculated by SpaceAngle (IMP-software).

Geometric morphometric analyses were performed using computer programs from the TPS series (TpsDig and TpsRegr), written by F.J. Rohlf (freely available at: http://life.bio.sunysb.edu/morph/) and the IMP series (CVAGen, DisparityBox, PCAGen, Regress6, Standard6, TwoGroup6, VecCompare, SpaceAngle), created by H.D. Sheets (freely available at: http://www2.canisius.edu/~sheets/morphsoft.html). TPS deformation grids were generated in the program MORPHEUS (Slice, 1999; http://www.morphometrics.org/morpheus.html. STATISTICA, version 8.1 (Statsoft 2005) was used for other statistical analyses (i.e., MANCOVA and MANOVA) and NMDS plots were generated using Matlab (The MathWorks 2007).

## Results

### Comparing ontogenetic allometries

The post-settlement ontogeny of the cephalic region is highly allometric in damselfishes. Shape variation in each skeletal unit is significantly correlated with log-transformed size (ln-CS) (all p levels of the Generalized Goodall's F test < 0.05, see Additional File 1) and a large proportion (up to 88%) of variation in total shape change during damselfish post-settlement development is explained by ontogenetic allometries.

The eight damselfishes show highly different allometric models. Indeed, interspecific differences in allometric models were highly significant for each structure (MANCOVA, Table 2). MANOVA revealed significant differences among species at the settlement stage (for each skeletal unit, p < 0.0001). All pairwise comparisons revealed statistically significant differences in larval shapes (*F*-tests, p < 0.0015); thus all species differ significantly from each even after Bonferroni adjustments for 26 comparisons. Moreover, the CVA assignment rates were high for each structure (from 91 to 98% depending to the skeletal unit).

**Table 2:** Tests for common linear allometric models in the eight damselfish species

*Element*	*λ_WILKS_*	*F*	*p*
*Neurocranium*	0.005	10.12	< 0.001
*Suspensorium and opercle*	0.063	6.59	< 0.001
*Mandible*	0.012	11.79	< 0.001
*Premaxilla*	0.114	13.50	< 0.001

Results of MANCOVA using Statistica 8.1.

**Figure 3:**
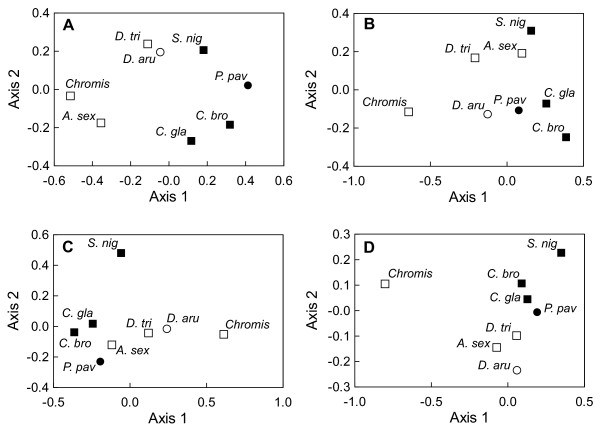
**Similarity of allometric trajectories**. Nonmetric multidimensional scaling plots on the matrix of pairwise angles among allometric trajectories for the (A) neurocranium, (B) the «suspensorium and opercle» unit, (C) the mandible and (D) the premaxilla. For each scatter plot, Kruskal Stress values are below 0.05. Different icons are used to illustrate the diet of each species: white square, zooplanktivorous; black square, algivorous; white circle, omnivorous feeding mainly on planktonic and benthic animal preys; black circle, omnivorous feeding mainly on filamentous algae. See Table 1 for abbreviations of the species.

The differences in the allometric models of damselfish species could also be attributed to their different rates in shape changes, or to their allometric patterns (i.e. the direction in which the ontogenetic trajectories point in the shape space), or to both. The analysis of the angles between multivariate regression vectors of ontogenetic allometries shows that the great majority of species have a species-specific allometric pattern for each skeletal unit (Table 3). Although belonging to the same genus, each angle between *C. glauca *and *C. brownriggii *was low but significantly different from zero (Table 3). On the other hand, the angle between *D. aruanus *and *D. trimaculatus *was not significantly different from zero for the neurocranium and the mandibule. The angle between *D. aruanus *and *P. pavo *was not significantly different from zero for the unit «suspensorium and opercle». Pairwise comparisons of angles between allometric vectors of each skeletal unit were summarized in the NMDS plots of Figure 3. The plot topology varied according to each skeletal unit although some constants were present. *Chromis *sp. is highly divergent from the other studied species for the majority of skeletal units, except for the neurocranium. According to the plot topology, a parallelism between the allometric patterns and the diet is present for the neurocranium, the mandible and the premaxilla (Figure 3A, C, D). Indeed, the first axis allowed good discrimination between mainly planktivorous and mainly algivorous species for the neurocranium and the mandible (Figure 3A, C). Concerning the premaxilla (Figure 3D), mainly algivorous species shared similar trajectories showing the highest values along axis 2 although *Chromis *sp seemed to be an exception. Finally, all large species (i.e. *A. sexfasciatus*, *D. trimaculatus*, *S. nigricans*; Table 1) showed similar allometric patterns for the unit «suspensorium and opercle».

**Table 3:** Comparisons between ontogenetic trajectories

		*A. sex*	*Chromis*	*C. bro*	*C. gla*	*D. aru*	*D. tri*	*P. pav*	*S. nig*
*Neurocranium*	*A. sex*	-							
	*Chromis*	**27.7**	-						
	*C. bro*	**45.4**	**53.4**	-					
	*C. gla*	**36.1**	**43.1**	**20.6**	-				
	*D. aru*	**33.5**	**38.5**	**38**	**37.4**	-			
	*D. tri*	**33.8**	**36.2**	**40.3**	**39.3**	12.6	-		
	*P. pav*	**47.1**	**53.6**	**28.5**	**32.5**	**37.3**	**40.3**	-	
	*S. nig*	**43.4**	**51.8**	**30.3**	**32.9**	**26**	**28.8**	**30.1**	-

*Suspensorium & opercle*	*A. sex*	-							
	*Chromis*	**45.5**	-						
	*C. bro*	**34.5**	**52.6**	-					
	*C. gla*	**25.6**	**41.4**	**20.6**	-				
	*D. aru*	**27.8**	**32.5**	**34.4**	**28.2**	-			
	*D. tri*	**26.3**	**31.9**	**42.1**	**29**	**24.9**	-		
	*P. pav*	**22.1**	**39.3**	**24.8**	**19.9**	17.2	**28**	-	
	*S. nig*	**22.8**	**50.2**	**32.5**	**26.6**	**30.5**	**26**	**28.9**	-

*Mandible*	*A. sex*	-							
	*Chromis*	**33.3**	-						
	*C. bro*	**18**	**40.5**	-					
	*C. gla*	**15.9**	**36.9**	**9.9**	-				
	*D. aru*	**21.8**	**20.1**	**28.9**	**24.2**	-			
	*D. tri*	**17.5**	**24.3**	**23.5**	**19.1**	8	-		
	*P. pav*	**16.8**	**33**	**17.9**	**17.8**	**21.9**	**20.3**	-	
	*S. nig*	**31.9**	**34.2**	**29.1**	**23.8**	**25.8**	**26**	**30.1**	-

*Premaxilla*	*A. sex*	-							
	*Chromis*	**72.9**	-						
	*C. bro*	**50.8**	**78.5**	-					
	*C. gla*	**40.8**	**89.6**	**18.7**	-				
	*D. aru*	**35.8**	**88.5**	**52.8**	**42.1**	-			
	*D. tri*	**27.1**	**75.2**	**39.1**	**31.8**	**23.4**	-		
	*P. pav*	**46.9**	**97.6**	**31.3**	**21.9**	**40.4**	**37.6**	-	
	*S. nig*	**58.2**	**101.3**	**50.6**	**46.4**	**59.1**	**55**	**43.4**	-

Angles between the ontogenetic vectors of the eight damselfish species are in decimal degrees. Results are obtained by bootstrapping procedure (N = 400) using VecCompare6. The angle between ontogenetic vectors is considered significant (in bold) if it exceeds the bootstrapped within-group variance at 95% confidence. See Table 1 for abbreviations of the species.

Using deformation grids, differences in allometric patterns among species are often visually striking (see Additional File 3). However, at large-scale, some ontogenetic shape changes are common to all species. Adults had shorter and higher neurocranium than larvae while these differences are limited in the two *Chrysiptera *species and *P. pavo*. In all other species, the heightening is mainly explained by the growth of the supraoccipital crest (LMs 5-7). Adults showed higher opercle and suspensorium than larvae. The suspensorium of adults is always shorter in its central part. Indeed, the distance between the articulation of the palatin (LM 2) and the two articulations of the hyomandibular (LMs 4, 5) is always shorter in adults. All larvae had a maxillary process of the palatin (LMs 1, 2) rostro-dorsally directed and an articulation quadrate-mandible (LM 8) behind a hypothetical vertical bar passing through the articulation of the palatin. The mandible of all larvae is less high showing a shorter symphisis mandibulae than the adult one. Except in *Chromis *sp., adults showed a shorter dentigerous process of the premaxilla (LMs 1, 6) than larvae. The ascending process of the premaxilla (LMs 1, 2) is always longer in adults.

**Figure 4:**
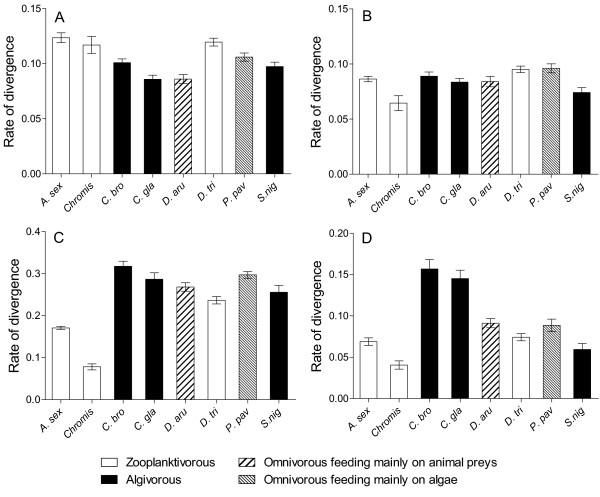
**Dynamics of shape changes**. (A) Neurocranium, (B) «suspensorium and opercle» unit, (C) mandible and (D) premaxilla. Rates of divergence (Mean ± Standard error) away from the larval shape, estimated by the regression of the Procrustes distance between each specimen and the average larval form of its species on log-transformed centroid size. The rates are expressed as the amount of shape changes per unit of size. The diet of each species is illustrated by patterned bars. See Table 1 for abbreviations of the species.

For each skeletal unit, the dynamic of shape changes (Procrustes distance Vs ln-CS) significantly differs among species (ANCOVA, Table 4). Figure 4 displays all rates of shape changes for each skeletal unit. Pairwise comparisons revealed that the developmental rate of the neurocranium was statistically significantly lower in *D. aruanus *and *C. glauca *than in the other species. Some significant differences existed among the other species but they cannot be subdivided into mutually exclusive subsets. Consequently we regard them as having the same developmental rate. The dynamic of shape changes varied little for the unit «suspensorium and opercle» although *Chromis sp. *and *S. nigricans *had the slowest rates, and *D. trimaculatus *and *P. pavo *the highest ones. The highest interspecific differences of developmental rates were observed for the mandible and the premaxilla in which *Chromis *sp had the significant lowest rate of shape changes for both units. The two *Chrysiptera *species had the highest developmental rates for the premaxilla. The rate of development of these two species was three times higher than the rate of *Chromis *sp. *Dascyllus aruanus *and *P. pavo *shared a lower developmental rate than both *Chrysiptera *but a higher one than *A. sexfasciatus*, *S. nigricans *and *D. trimaculatus*. For the mandible, *A. sexfasciatus *had a rate of shape change higher than *Chromis *sp but lower than the developmental rates of the other species. In conclusion, the dynamics of shape change were not very different among species and there was little evidence of a hypothetical relationship between the rate of shape changes and the diet of the species (Figure 4). For the neurocranium, the unit «suspensorium and opercle», the mandible and the premaxilla, all studied species can be subdivided in 2, 3, 3 and 4 groups, respectively. No correlation existed between the rate of development of a skeletal unit and the pelagic larval duration (PLD) of the species (0.52 < p < 0.95 according to the skeletal unit), nor between the developmental rate and the size variation undergone during the post-settlement growth of the species (0.12 < p < 0.83 according to the skeletal unit).

**Table 4:** Tests for differences in developmental rates in the eight damselfish species

*Element*	*F*	*df*	*p*
*Neurocranium*	13.50	7, 368	< 0.001
*Suspensorium and opercle*	6.66	7, 382	< 0.001
*Mandible*	57.34	7, 385	< 0.001
*Premaxilla*	26.96	7, 382	< 0.001

Results of ANCOVA using Statistica 8.1.

**Figure 5:**
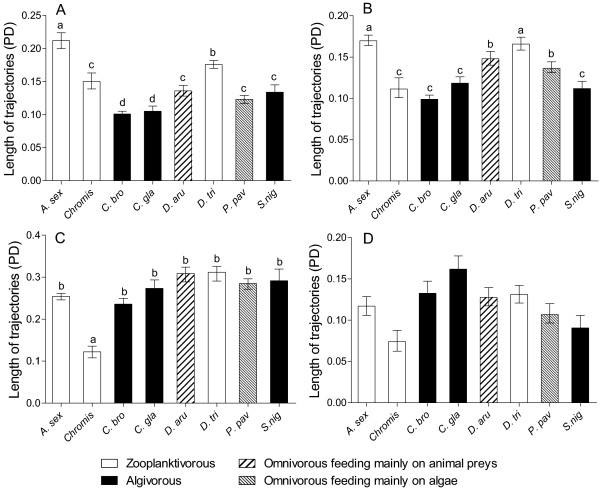
**Lengths of ontogenetic trajectories**. (A) Neurocranium, (B) «suspensorium and opercle» unit, (C) mandible and (D) premaxilla. Length of the trajectories in units of Procrustes distance for each skeletal unit (Mean with 95% CI). Confidence limits are obtained by bootstrapping procedure (N = 400). The diet of each species is illustrated by patterned bars. See Table 1 for abbreviations of the species.

The length of ontogenetic trajectories differs among damselfishes for each skeletal unit (Figure 5). These lengths, expressed in Procrustes distance, are directly proportional to the amount of shape changes undergone by every species during growth. For the neurocranium, *A. sexfasciatus *showed the longest ontogenetic trajectory and the two *Chrysiptera *species had the shortest ones. According to the confidence limits (95% CI) obtained by the bootstrapping procedure, *Chromis *sp, *D. aruanus*, *P. pavo *and *S. nigricans *could be differentiated statistically from each other in pairwise comparisons, but they cannot be subdivided into mutually exclusive subsets by length. So we regard them as having the same length. *Dascyllus trimaculatus *had an intermediate length of ontogenetic trajectory between these four species and *A. sexfasciatus*. For the unit «suspensorium and opercle», the lengths of the ontogenetic trajectory of *A. sexfasciatus *and *D. trimaculatus *were significantly longer than the other species. On the other hand, *Chromis *sp, the two *Chrysiptera *species and *S. nigricans *underwent the lowest amount of shape changes during growth. The length of the ontogenetic trajectory of *Chromis *sp was almost twice as short as the other seven studied species. *Dascyllus aruanus *and *P. pavo *had an intermediate length. *Chromis *sp had the shortest length of ontogenetic trajectory for the mandible. For both mouthparts, all other species did not show significant differences in the length of ontogenetic trajectories. In conclusion, these comparisons suggest that the length of ontogenetic trajectory is a moderately variable developmental trait. Indeed, all studied species can be subdivided into 4, 3, 2 and 1 group(s) for the neurocranium, the unit «suspensorium and opercle», the mandible and the premaxilla, respectively. The amount of shape changes undergone by each species is not correlated to the size variation observed during the post-settlement growth, except for the neurocranium (neurocranium *r *= 0.84, p < 0.01; suspensorium and opercle *r *= 0.66, p = 0.07; mandible *r *= 0.25, p = 0.55; premaxilla *r *= -0.16, p = 0.70). No correlation existed between the length of ontogenetic trajectory and the mean PLD of species (0.26 < p < 0.83 according to the skeletal units).

### Comparing levels and patterns of shape disparity at three ontogenetic stages

**Figure 6:**
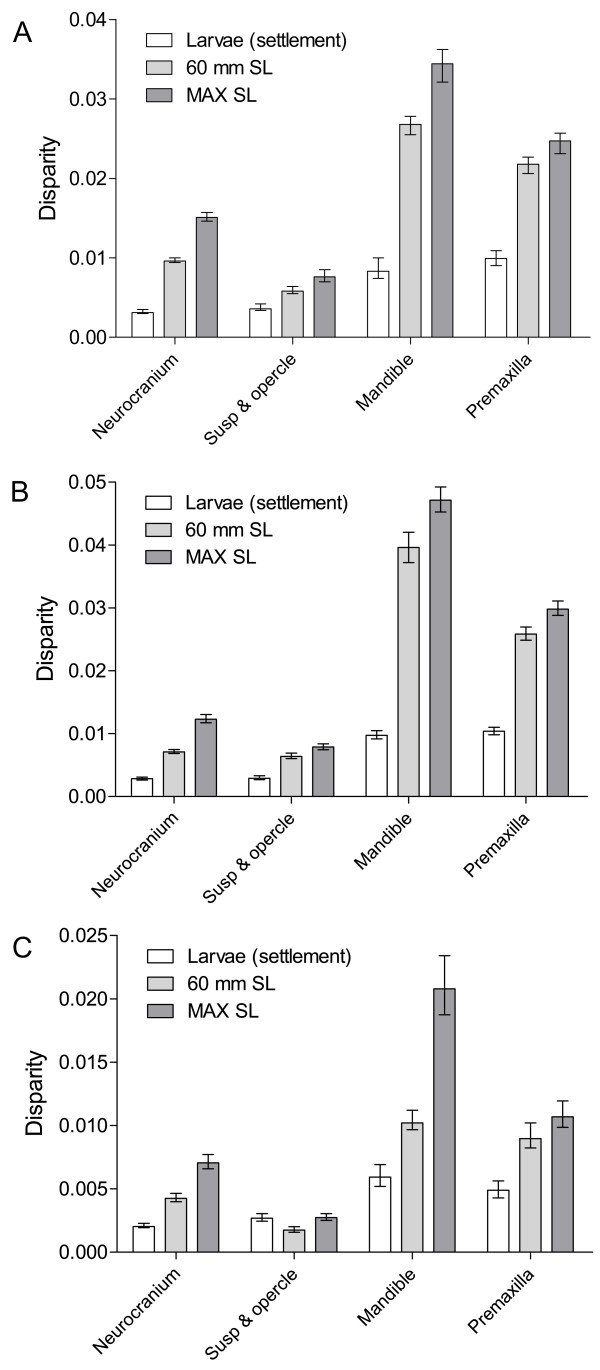
**Ontogenetic dynamics of shape disparity over ontogeny among all studied species and within each main trophic group**. Shape disparity (Mean with 95% CI) at the three studied ontogenetic stages: at settlement, at 60 mm SL and at the maximum SL (MAX) for (A) the family level, within (B) mainly zooplanktivorous and (C) mainly algivorous species. Confidence limits are obtained by bootstrapping procedure (N = 400).

The shape disparity among species significantly increased over ontogeny for each skeletal unit (Figure 6A). The neurocranium and the mandible showed the most spectacular variation. Indeed, the neurocranium and the mandible shapes were respectively five and four times more disparate at the adult stage (MAX SL) than at the larval stage (settlement). The increase of shape disparity is the lowest for the unit «suspensorium and opercle». Calculation of the levels of shape disparity at an intermediate size, common for each studied species, revealed differences in the ontogenetic dynamic of disparity among structures. The level of shape disparity for the neurocranium in fishes sizing 60 mm SL accounts for 64% of the level of disparity at the MAX SL while, at the same intermediate size, the disparity of the premaxilla is 88% of the maximal disparity. The two other skeletal units show intermediate values: the disparity level of the unit «suspensorium and opercle» and the mandible at 60 mm SL is 76% and 78% of the maximal disparity, respectively.

The shape disparity significantly increased over ontogeny within both main trophic groups (i.e. mainly zooplanktivorous and mainly algivorous species, Figure 6B-C). Within each trophic group, the increasing of disparity is most important for the neurocranium, the mandible and the premaxilla. Conversely to zooplanktivorous species, the group of mainly algivorous species did not show significant variation of shape disparity of the unit «suspensorium and opercle» over ontogeny.

**Figure 7:**
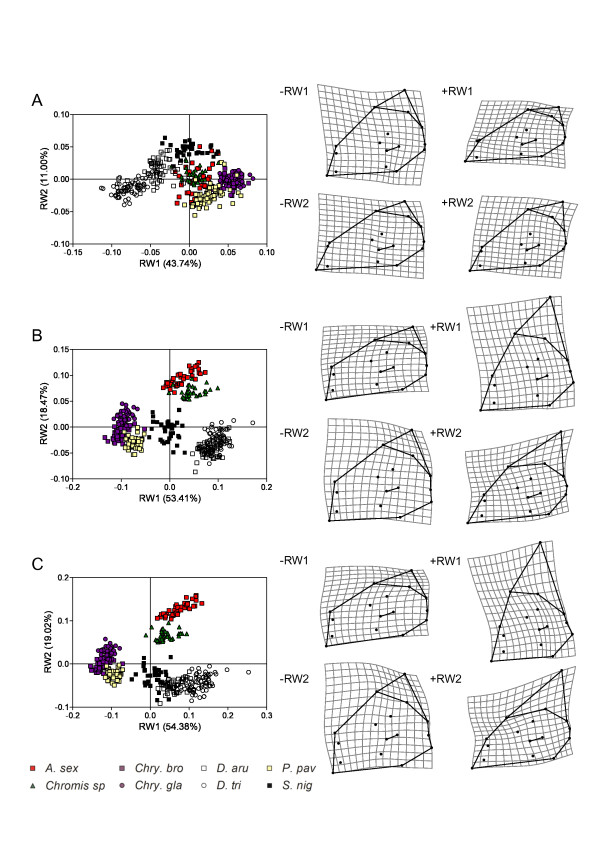
**Ontogenetic dynamics of the patterns of neurocranium shape disparity**. Relative warps analyses of the neurocranium shapes at the three ontogenetic stages. A, shapes at the settlement stage. B, shapes at an intermediate size of 60 mm SL. C, shapes at the maximum adult body size. TPS deformation grids indicate shape variation represented by RW1 and RW2 (minimal (-RW) and maximal (+RW) values).

**Figure 8:**
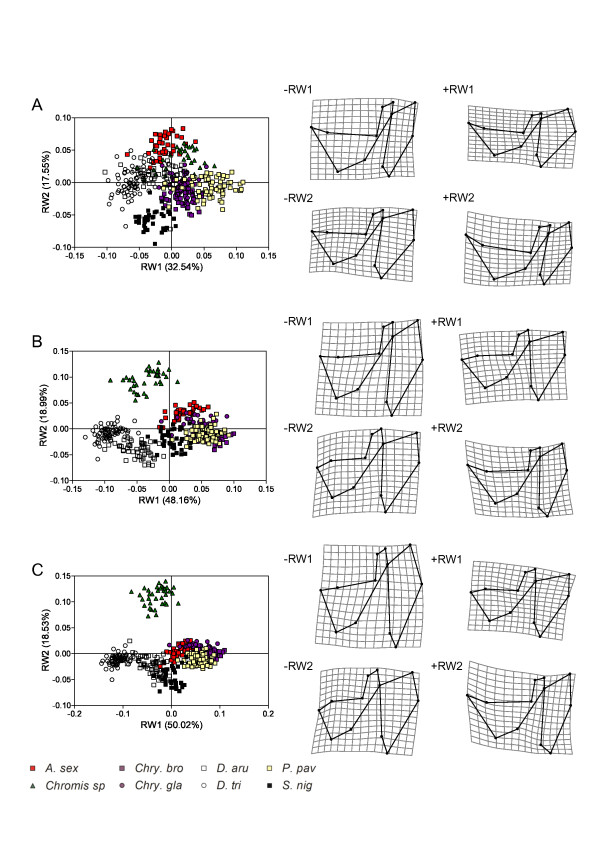
**Ontogenetic dynamics of the patterns of suspensorium and opercle shape disparity**. Relative warps analyses of the suspensorium and opercle shapes at the three ontogenetic stages. A, shapes at the settlement stage. B, shapes at an intermediate size of 60 mm SL. C, shapes at the maximum adult body size. TPS deformation grids indicate shape variation represented by RW1 and RW2 (minimal (-RW) and maximal (+RW) values).

**Figure 9:**
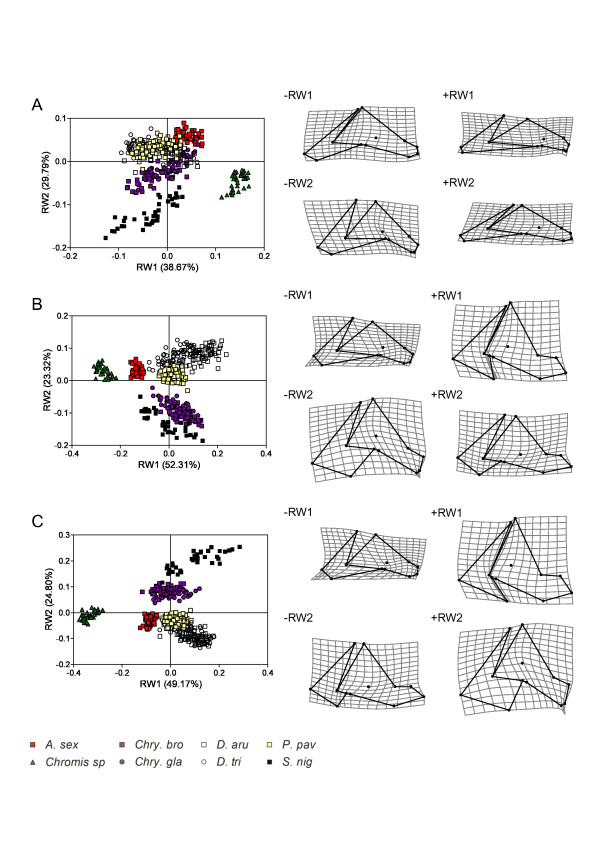
**Ontogenetic dynamics of the patterns of mandible shape disparity**. Relative warps analyses of the mandible shapes at the three ontogenetic stages. A, shapes at the settlement stage. B, shapes at an intermediate size of 60 mm SL. C, shapes at the maximum adult body size. TPS deformation grids indicate shape variation represented by RW1 and RW2 (minimal (-RW) and maximal (+RW) values).

**Figure 10:**
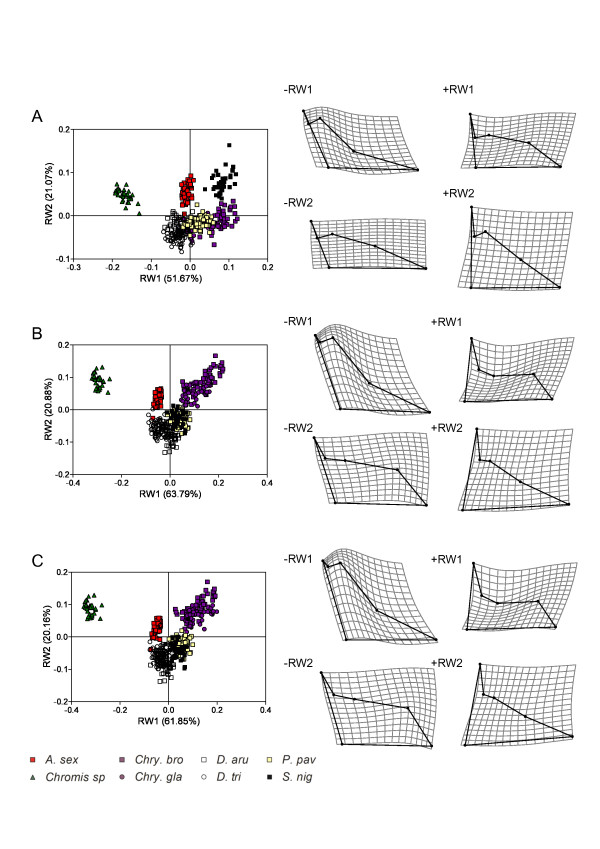
**Ontogenetic dynamics of the patterns of premaxilla shape disparity**. Relative warps analyses of the premaxilla shapes at the three ontogenetic stages. A, shapes at the settlement stage. B, shapes at an intermediate size of 60 mm SL. C, shapes at the maximum adult body size. TPS deformation grids indicate shape variation represented by RW1 and RW2 (minimal (-RW) and maximal (+RW) values).

The patterns of shape disparity also varied throughout ontogeny. The distribution of shapes in the plane defined by the first two relative warps shows that larvae and adults do not occupy the same sub-space of shape spaces (Figures 7, 8, 9, 10), indicating a redistribution of variance over ontogeny.

At settlement, the first two relative warps RW1 and RW2 explain 54.7% of the total shape variance of the neurocranium (Figure 7). Having the lowest scores on the RW1 axis, the two *Dascyllus *species showed a higher neurocranium than the others. The second component (RW2), explaining 11% of the shape variance, highlights small differences in the shape of the supraoccipital crest and in the relative length of the orbital and the post-orbital regions. The variance explained by RW1 and RW2 is higher at the size of 60 mm SL and at the maximum adult body size (~72% of total shape variance) (Figure 7). Three groups can be easily distinguished along the RW1 axis at 60 mm, a first including the two *Chrysiptera *species and *P. pavo*, a second grouping *A. sexfasciatus*, *Chromis *sp and *S. nigricans*, and a third including the two *Dascyllus *species. *Pomacentrus pavo *and both *Chrysiptera *have a proportionally longer neurocranium. *Abudefduf sexfasciatus *and *Chromis *sp show a larger supraoccipital crest than the other species of the second group (RW2). At the maximum size, *S. nigricans *shares a more similar shape to the two *Dascyllus *species allowing the clear distinction of three groups in the shape space defined by RW1 and RW2. The two *Chrysiptera *species, *P. pavo *and *S. nigricans*, have a shorter and ventrally directed vomer (low RW1 and RW2 scores, see LMs 1-2).

The shape variance of the unit «suspensorium and opercle» is relatively low at the settlement stage (Figure 8). The two *Dascyllus *species have the lowest RW1 values and *P. pavo *the highest. Both *Dascyllus *species show the narrowest opercle and the highest unit «suspensorium and opercle». The second component (RW2) opposes *S. nigricans*, which shows a horizontal maxillary process of the palatin, to *A. sexfasciatus *and *Chromis *sp., in which this process is rostro-dorsally oriented. At 60 mm, the first component (RW1) directly opposes the two *Dascyllus *species to the two *Chrysiptera *species and *P. pavo*; *A. sexfasciatus *and *S. nigricans *occupy an intermediate position (Figure 8). The second component (RW2) allows the discrimination of *Chromis *sp. having a lower suspensorium and a maxillary process rostro-dorsally oriented. At the maximum size, *S. nigricans *has a ventrally bent maxillary process of the palatin (-RW2) (Figure 8).

At the settlement stage, the first two components explain nearly equal amounts of variance of mandible shape (RW1 = 39% and RW2 = 30%) (Figure 9). Only *Chromis *sp. and *S. nigricans *show distinct shapes from the other species. *Chromis *sp. has the highest RW1 scores, revealing a lower mandible with a short retroarticular. *Stegastes nigricans *differs from the others along RW2, showing a shorter angular in its ventral part. At 60 mm and maximum body size, the first two relative warps seem to express almost the same percentage of total shape variance and the same pattern of shape variation (Figure 9). For both stages, RW1 (~ 50% of the total shape variance) mainly differentiates species according to the height of the mandible and the length of the symphisis mandibulae, distinguishing *A. sexfasciatus *and *Chromis *sp. from the other species. The second relative warp (24% of the shape variance) distinguishes the three herbivorous species (the two *Chrysisptera *and *S. nigricans) *from *P. pavo *and the two *Dascyllus *by their massive mandible (Figure 9).

The dynamic of the pattern of premaxilla shape disparity seems similar to that of the mandible (Figure 10). At settlement stage, *Chromis *sp. shows a highly divergent shape along RW1 revealing a thin dentigerous process and an obtuse angle between the ascending and the dentigerous processes. The second relative warp distinguishes *A. sexfasciatus *and *S. nigricans *from the two *Chrysiptera*, the two *Dascyllus *species and *P. pavo*. Indeed, the first ones have a shorter dentigerous process and a longer ascending process than the others. The pattern of shape disparity is the same for the intermediate and the adult stages (Figure 10). *Chromis *sp. is highly divergent from all other species along RW1; and *A. sexfasciatus *and the two *Chrysiptera *species are distinguished from the two *Dascyllus *species, *S. nigricans *and *P. pavo *along RW2.

The calculation of the angles among sub-spaces defined by the first two relative warps corroborates the previous visual explorations (Table 5): larvae and adults occupy different sub-spaces of morphospaces. For the neurocranium, the angle between the sub-spaces of the settlement stage and the maximum adult body size is 86° and thus largely higher than the ranges of the within-hyperplane angles (64° for the settlement stage and 14° for the maximum size). Consequently, these hyperplanes are significantly distinct from each other in the total shape space, revealing that the pattern of shape disparity of larvae at settlement differs from that of adults. The same conclusions can be drawn for (1) all comparisons between the hyperplanes of settlement stage and maximum adult size and for (2) all comparisons between the hyperplanes of settlement stage and intermediate stage (60 mm) for each skeletal unit (Table 5). On the other hand, the results differ for the comparisons between the hyperplanes of 60 mm stage and maximum size stage according to the skeletal unit, highlighting differences in the dynamics of the patterns of shape disparity. For the neurocranium and the unit «suspensorium and opercle», the analyses show that the shapes at 60 mm and at the maximum adult body size occupy different sub-spaces. Conversely, the angle between these hyperplanes is not significant for the mandible and the premaxilla (Table 5); thus the patterns of shape disparity are similar at these two ontogenetic stages for both skeletal units.

**Table 5:** Comparisons between hyperplanes of skeletal shapes

*Element*	*Stages 1-2*	*Within-stage 1*	*Within-stage 2*	*Between-stages*	*Significance*
*Neurocranium*	Larvae-60 mm	70.06	10.75	79.83	S
	Larvae-MAX	64.28	14.01	86.39	S
	60 mm-MAX	10.38	14.08	15.52	S
*Susp. & opercle*	Larvae-60 mm	36.23	12.9	55.6	S
	Larvae-MAX	35.93	13.46	56.54	S
	60 mm-MAX	13.29	13.03	16.68	S
*Mandible*	Larvae-60 mm	10.77	12.72	44.54	S
	Larvae-MAX	12.47	20.89	44.32	S
	60 mm-MAX	12.41	18.6	16.04	NS
*Premaxilla*	Larvae-60 mm	21.03	6.67	61.75	S
	Larvae-MAX	20.24	8.6	70.38	S
	60 mm-MAX	6.62	9.19	9.04	NS

Comparing hyperplanes of skeletal shape variation between the three studied ontogenetic stages (larvae at settlement, 60 mm SL and maximum SL). Results are obtained by bootstrapping procedure (N = 400) using SpaceAngle. Angles are in decimal degrees. The angle between hyperplanes is considered significant (S) if it exceeds the bootstrapped within-group variance at 95% confidence.

## Discussion

The level and the pattern of head skeleton shape disparity significantly vary throughout the post-settlement ontogeny of damselfishes. The disparity level increases over ontogeny both at the family level and within each trophic group (Figure 6). Adults are more disparate in shapes and occupy different sub-spaces of the total shape spaces than settling larvae, implying reorganization of variance with growth. Although larval shapes are more similar, they are already species-specific at the time of the coral reef settlement probably due to differences in the larval growth and/or the pelagic larval duration (Table 1). Indeed, all species do not have the same age when they settle on the coral reef [58].

According to the study of developmental parameters, the increasing disparity seems to be mainly due to the divergence of allometric patterns. This parameter is clearly not constrained: nearly every species has their own allometric pattern for each skeletal unit (Table 3). Eight specific allometric patterns exist for the premaxilla and 7 for the other units (Table 6). In the shape space, every species has its own ontogenetic trajectory. Only the allometric vectors of the neurocranium and the mandible in *D. aruanus *and *D. trimaculatus *point in the same direction (Table 3). Both species are dissimilar at each stage (i.e. larvae and adult) but have parallel ontogenetic trajectories in the size-shape space, illustrating a case of lateral transposition where dissociation had occurred in an earlier period of reef settlement. The conservation of the same allometric pattern in these two closely related species belonging to the same genus may be explained by genetic similarities. More surprisingly, the allometric patterns of the unit «suspensorium and opercle» in *D. aruanus *and *P. pavo *reveal a third case of parallel trajectories. All others show a total divergence of allometric trajectories (i.e. allometric repatterning). Generally speaking, the range of the divergence of allometric patterns (9.9°-53.6°, Table 3) is similar to the results obtained from other geometric morphometric studies of different structures of the head skeleton in vertebrates [31,33,35,59,60], except for the premaxilla which is highly divergent (18.7°-101.3°). The angles among the ontogenetic trajectories seem to be fairly related to the diet (Figure 3). On the other hand, the incongruence between allometric trajectories and phylogeny (Figures 1 &3) may be linked to the reticulate adaptive radiation (i.e. evolutionary patterns characterized by rapid and repeated shifts between a limited number of ecomorphological states) of damselfishes suggested by Cooper and Westneat [3]. Indeed, our data probably illustrate successive allometric repatternings allowing ecomorphological shifts during the evolution of Pomacentridae. This result implied that the ontogenetic trajectories of the damselfish species are evolutionarily labile. The evolutionary changes seen in ontogenetic trajectories across the phylogeny may reflect the complex interplay between developmental processes and selective pressure that are more intense at various developmental stages (e.g. reef settlement stage and/or adult stage) [19]. The variability of the angles within the same genus (e.g. *Chrysiptera *and *Dascyllus*) remains limited in spite of some differences according to the species and the skeletal units. In the two *Chrysiptera *species, the divergence of allometric trajectories is low for every studied structure. On the other hand, the two *Dascyllus *species show two identical allometric patterns. Likewise the phylogenetic proximity of the genera *Pomacentrus *and *Chrysiptera *suggested by molecular data (Figure 1) is confirmed by the similarity of allometric trajectories. Finally, a functional demand related to the head size could explain the similarity of allometric patterns for the unit «suspensorium and opercle» among the large species: *D. trimaculatus, A. sexfasciatus *and *S. nigricans *(Figure 3).

**Table 6:** Diversity of developmental parameters observed in the eight studied species

*Element*	*Larval shapes*	*Allometric patterns*	*Rates of development*	*Lengths of ontogenetic trajectories*
*Neurocranium*	8	7	2	4
*Susp. & opercle*	8	7	3	3
*Mandible*	8	7	3	2
*Premaxilla*	8	8	4	1

The figures represent the diversity of studied allometric parameters (i.e. larval shapes, allometric trajectories, rates of shape changes and lengths of allometric trajectory) among damselfishes. As eight damselfish species were studied, 8 is a maximum stating that each species has a specific developmental parameter.

The length of ontogenetic trajectory and the rate of shape change appear to be conservative developmental parameters. Depending on the skeletal unit studied, 1 to 4 values have been highlighted for 8 species (Table 6). Thus some species share the same length of ontogenetic trajectory or the same dynamics of shape change. However the variation of both parameters could be underestimated by our methodology. Indeed, having no age information on the studied specimens, compensatory changes in growth rate (defined as the relationships between age and size) could not be explored. These two developmental parameters are not correlated to the duration of the larval phase or the size variation of post-settlement ontogeny. The reasons for such a low variability of these parameters are not obvious. During settlement, the great majority of damselfishes directly recruit to the adult populations and thus directly use the same habitat of their congeners [61,62] unlike other taxa which undergone one or some habitat changes before their recruitment (e.g. Labridae, Acanthuridae, Serranidae). Among the studied species, only *A. sexfasciatus *deviates from this general trend: the juveniles settle in micro-atolls of the fringing reef and adults live on the barrier reef [61]. This consistent behavior pattern observed in pomacentrids could partially explain the low inter-specific variability of the rate of development. Both parameters are not related to diet and phylogeny. Intuitively, we hypothesized that planktivorous species at the adult stage, thus having no ontogenetic change of diet, undergo lower amount of shape changes than herbivorous species but that is not verified. The ontogenetic shape changes that are related to a shift of feeding strategies [38,50] and other functional demands might also explain shape changes related to respiration [63] or sound production [64].

**Figure 11:**
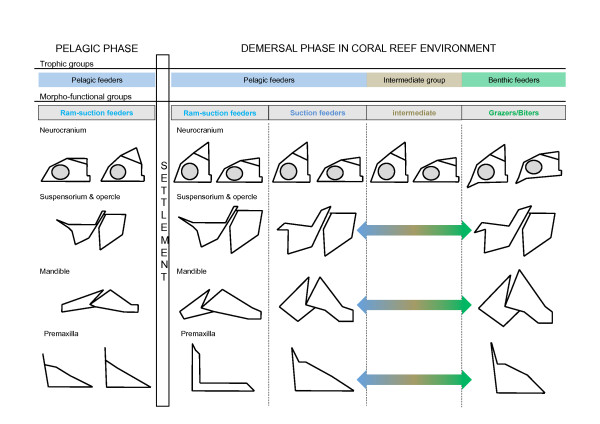
**Schematic representation of trophic diversity and morpho-functional disparity throughout ontogeny of damselfishes**. See the text for details. Arrows express gradient of shapes between the two extremes of morpho-functional group.

Allometry is an important factor in modeling of the head shape during the post-settlement growth of damselfishes. Our results reveal a higher degree of similarity at reef settlement, followed by a significant spreading of species within morphospace over ontogeny. Different factors could explain a closer similarity among settling larvae than among adults, including the internal (e.g. functional and developmental) and external (e.g. ecological) constraints. The latter might seem predominant. Indeed, as all larvae live in the pelagic zone of the ocean [13], the shape similarity seems to be linked to a common environment and very similar diets, exclusively composed of copepod nauplii and adults [27]. On the other hand, the lagoon and the barrier reef offer a multitude of resources and feeding habits. Three major modes of feeding have been identified in Teleost fishes: sucking, biting and ram feeding (for a review see [38]). These three modes may be predicted from the functional design of the damselfish head [3,4] although they possess skulls of intermediate design that will also allow some opportunism in their diet [28,65]. The allometric patterns detailed in the present study strengthen previous data revealing an improvement of suction feeding system during damselfish ontogeny [50]. The main allometric shape changes include: a heightening of the suspensorium and the opercle, an elevation of the supraoccipital crest, a forward displacement of the mandibular articulation with respect to the neurocranium, a mandible becoming proportionally shorter and a lengthening of the ascending process of the premaxilla. Figure 8 summarizes the variation of trophic diversity and morphological disparity throughout ontogeny of damselfishes, highlighted during the present study and others [4,28,29,50,66,67]. As demonstrated in other Teleosts such as Labridae [68,69] and Centrarchidae [70], morphological (shape) diversity may not always be an accurate predictor of biomechanical diversity. An overview of our previous studies dealing with feeding habits [28] and trophic morphology [4,66,67] in adult damselfishes shows that the higher level of shape disparity corresponds to a higher level of ecological and functional diversity in comparison to the larval stage. Nevertheless, future studies will have to specifically test the relationship between the morphological (shape) disparity and the mechanical disparity in damselfishes, as done by Hulsey and Wainwright [71] in labrids. Although larval shapes are already species-specific, some similarities suggest that settling larvae can be considered as ram-suction feeders (see discussion in [50]). Adult damselfishes show either a short neurocranium type (*S. nigricans*, *A. sexfasciatus*, *Chromis *sp, both *Dascyllus *species) or a more lengthened type (both *Chrysiptera *species, *P. pavo*). The main character allowing the differentiation of grazing species is the presence of a ventrally bent vomerian region (Figure 11). The other skeletal shapes literally vary between the two main functional constraints: suction feeding and grazing/biting (Figure 11). The pelagic feeders (*A. sexfasciatus *and *D. trimaculatus*) and the benthic feeders (both *Chrysiptera *species and *S. nigricans*) show skeletal shapes optimizing suction feeding (e.g. high suspensoria and opercles, short mandible) and grazing (e.g. robust mandible, short and massive dentigerous process of the premaxilla, ventrally bent maxillary process of the palatin, broad hyomandibular allowing a large place for the muscle adductor mandibulae) respectively [3,4]. Species belonging to the intermediate group such as *P. pavo *have intermediate skeletal shapes or their head is a mix of skeletal units typical of suction feeding or grazing species. Among the pelagic feeders, *Chromis *sp (i.e. *Chromis viridis *and *C. atripectoralis*, see Materials & Methods) showed some striking divergences (Figures 3, 7, 8, 9, 10). The shapes and the short lengths of ontogenetic trajectories (Figure 4) are suggestive of functional similarity with larvae. Furthermore our morphological and developmental data corroborate kinematic studies [72] showing that *Chromis viridis *use ram-suction feeding to capture highly evasive prey such as copepods. In summary, one main morpho-functional group can be recognized at the pelagic larval stage and three at the demersal adult stage (Figure 11).

## Conclusion

The present study describes qualitatively and quantitatively the diversification of the head skeleton during the post-settlement growth of damselfishes. The morphological disparity of the head skeleton is higher at the adult stage associated with the coral reef environment in comparison with the settling larval stage. At reef settlement, larval shapes are already species-specific probably due to differences in age. The process of diversification throughout the ontogeny of damselfishes is mainly explained by the divergence of allometric trajectories in shape space. The length of ontogenetic trajectory and the rate of development seem to be more constrained developmental parameters. The bipartite life-cycle of damselfishes exemplifies a case where the environment (i.e. the coral reef) promotes the increasing shape disparity of the head skeleton over ontogeny. Linked to different developmental parameters, we show adult head morphology diverged on various phenotypes across species, suggesting that functional demands for varied feeding strategy are more intense in adults than in larvae. Our study demonstrates that both selection and developmental processes have influenced phenotypic evolution in this fish group.

## Authors' contributions

BF and PV both contributed to the collection of pomacentrid specimens. BF and PV conceived of the study. BF carried out fish staining, dissections and morphometric analyses; analyzed the data; and wrote the manuscript. All authors read and approved the final manuscript.

## Supplementary Material

Additional File 1 **Fit of regressions of shape versus log-values of centroid size (CS) for each skeletal unit**. Tabular data (.xls). Results of regression analyses using TPSRegr (Version 1.34). Click here for file

Additional File 2 **Plots of Procrustes Distance (PD) between each specimen and the average shape of larvae on log-transformed centroid size (ln-CS)**. Example of *Abudefduf sexfasciatus*, *Dascyllus aruanus *and *Pomacentrus pavo *for each studied skeletal unit: the neurocranium, the unit «suspensorium and opercle», the mandible and the premaxilla. Click here for file

Additional File 3 **Illustrations of ontogenetic shape changes**. TPS deformation grids illustrating the ontogenetic shape changes for each skeletal unit in every studied species Click here for file

## References

[B1] Woods PJ Habitat-dependent geographical variation in ontogenetic allometry of the shiner perch *Cymatogaster aggregata *Gibbons (Teleostei: Embiotocidae) Journal of Evolutionary Biology 2007 20 5 1783 1798 10.1111/j.1420-9101.2007.01386.x 17714296

[B2] Bellwood DR Wainwright PC Fulton CJ Hoey AS Functional versatility supports coral reef biodiversity Proceedings of the Royal Society B-Biological Sciences 2006 273 1582 101 107 10.1098/rspb.2005.3276 PMC1560014 16519241

[B3] Cooper WJ Westneat MW Form and function of damselfish skulls: rapid and repeated evolution into a limited number of trophic niches Bmc Evolutionary Biology 2009 9 10.1186/1471-2148-9-24 19183467 PMC2654721

[B4] Frédérich B Pilet A Parmentier E Vandewalle P Comparative trophic morphology in eight species of damselfishes (Pomacentridae) Journal of Morphology 2008 269 2 175 188 17935195 10.1002/jmor.10586

[B5] Fulton CJ Bellwood DR Wainwright PC Wave energy and swimming performance shape coral reef fish assemblages Proceedings of the Royal Society B-Biological Sciences 2005 272 1565 827 832 10.1098/rspb.2004.3029 PMC1599856 15888415

[B6] Konow N Bellwood DR Wainwright PC Kerr AM Evolution of novel jaw joints promote trophic diversity in coral reef fishes Biological Journal of the Linnean Society 2008 93 3 545 555 10.1111/j.1095-8312.2007.00893.x

[B7] Motta PJ Functional morphology of the feeding apparatus of ten species of Pacific butterflyfishes (Perciformes, Chaetodontidae): an ecomorphological approach Environmental Biology of Fishes 1988 22 1 39 67 10.1007/BF00000543

[B8] Riedlecker E Herler J Trophic morphology of the coral-associated genus Gobiodon (Teleostei: Gobiidae) from the Red Sea Journal of Zoological Systematics and Evolutionary Research 2009 47 2 160 170 10.1111/j.1439-0469.2008.00497.x

[B9] Wainwright PC Bellwood DR Sale PF Ecomorphology of feeding in coral reef fishes Coral reef fishes: dynamics and diversity in a complex ecosystem 2002 San Diego: Academic Press 33 56

[B10] Wainwright PC Bellwood DR Westneat MW Grubich JR Hoey AS A functional morphospace for the skull of labrid fishes: patterns of diversity in a complex biomechanical system Biological Journal of the Linnean Society 2004 82 1 1 25 10.1111/j.1095-8312.2004.00313.x

[B11] Fulton CJ Bellwood DR Ontogenetic habitat use in labrid fishes: an ecomorphological perspective Marine Ecology-Progress Series 2002 236 255 262 10.3354/meps236255

[B12] Wainwright PC Richard BA Predicting patterns of prey use from morphology of fishes Environmental Biology of Fishes 1995 44 1-3 97 113 10.1007/BF00005909

[B13] Leis JM Sale PF The pelagic stage of reef fishes The ecology of fishes on coral reefs 1991 San Diego: Academic Press 183 230

[B14] Bellwood DR Wainwright PC Sale PF The history and biogeography of fishes on coral reefs Coral Reef Fishes: Dynamics and Diversity in a Complex Ecosystem 2002 London: Academic Press 5 32

[B15] Erwin DH Disparity: Morphological pattern and developmental context Palaeontology 2007 50 57 73 10.1111/j.1475-4983.2006.00614.x

[B16] Holtmeier CL Heterochrony, maternal effects, and phenotypic variation among sympatric pupfishes Evolution 2001 55 2 330 338 11308091 10.1554/0014-3820(2001)055[0330:HMEAPV]2.0.CO;2

[B17] Loy A Bertelletti M Costa C Ferlin L Cataudella S Shape changes and growth trajectories in the early stages of three species of the genus Diplodus (Perciformes, Sparidae) Journal of Morphology 2001 250 1 24 33 10.1002/jmor.1056 11599013

[B18] Zelditch ML Sheets HD Fink WL The ontogenetic dynamics of shape disparity Paleobiology 2003 29 1 139 156 10.1666/0094-8373(2003)029<0139:TODOSD>2.0.CO;2

[B19] Adams DC Nistri A Ontogenetic convergence and evolution of foot morphology in European cave salamanders (Family: Plethodontidae) BMC Evolutionary Biology 2010 10 216 10.1186/1471-2148-10-216 20637087 PMC2927916

[B20] Ciampaglio CN Determining the role that ecological and developmental constraints play in controlling disparity: examples from the crinoid and blastozoan fossil record Evolution & Development 2002 4 3 170 188 10.1046/j.1525-142x.2002.02001.x 12054291

[B21] Foote M Morphological disparity in ordovician-devonian crinoids and the early saturation of morphological space Paleobiology 1994 20 3 320 344

[B22] Foote M Morphological diversification of paleozoic crinoids Paleobiology 1995 21 3 273 299

[B23] Eble GJ Contrasting evolutionary flexibility in sister groups: disparity and diversity in Mesozoic atelostomate echinoids Paleobiology 2000 26 1 56 79 10.1666/0094-8373(2000)026<0056:CEFISG>2.0.CO;2

[B24] Hall BK Bauplane, phylotypic stages, and constraint - Why there are so few types of animals Evolutionary Biology, Vol 29 1996 29 215 261

[B25] Allen GR Damselfishes of the world 1991 Melle: Publication of natural history and pets book, Mergus

[B26] Cooper WJ Smith LL Westneat MW Exploring the radiation of a diverse reef fish family: Phylogenetics of the damselfishes (Pomacentridae), with new classifications based on molecular analyses of all genera Molecular Phylogenetics and Evolution 2009 52 1 1 16 10.1016/j.ympev.2008.12.010 19135160

[B27] Sampey A McKinnon AD Meekan MG McCormick MI Glimpse into guts: overview of the feeding of larvae of tropical shorefishes Marine Ecology-Progress Series 2007 339 243 257 10.3354/meps339243

[B28] Frédérich B Fabri G Lepoint G Vandewalle P Parmentier E Trophic niches of thirteen damselfishes (Pomacentridae) at the Grand Récif of Toliara, Madagascar Ichthyological Research 2009 56 1 10 17

[B29] Emery AR Comparative ecology and functional osteology of fourteen species of damselfish (Pisces: Pomacentridae) at Alligator Reef, Florida Keys Bulletin of Marine Science 1973 23 649 770

[B30] Webster M Ontogeny and evolution of the early Cambrian trilobite genus Nephrolenellus (Olenelloidea) Journal of Paleontology 2007 81 1168 1193 10.1666/06-092.1

[B31] Ivanovic A Vukov TD Dzukic G Tomasevic N Kalezic ML Ontogeny of skull size and shape changes within a framework of biphasic lifestyle: a case study in six Triturus species (Amphibia, Salamandridae) Zoomorphology 2007 126 3 173 183 10.1007/s00435-007-0037-1

[B32] Monteiro LR Cavalcanti MJ Sommer HJS Comparative ontogenetic shape changes in the skull of Caiman species (Crocodylia, Alligatoridae) Journal of Morphology 1997 231 1 53 62 10.1002/(SICI)1097-4687(199701)231:1<53::AID-JMOR5>3.0.CO;2-P 29852629

[B33] Cardini A O'Higgins P Post-natal ontogeny of the mandible and ventral cranium in Marmota species (Rodentia, Sciuridae): allometry and phylogeny Zoomorphology 2005 124 4 189 203 10.1007/s00435-005-0008-3

[B34] Collard M O'Higgins PO Ontogeny and homoplasy in the papionin monkey face Evolution & Development 2001 3 5 322 331 10.1046/j.1525-142x.2001.01042.x 11710764

[B35] Bastir M O'Higgins P Rosas A Facial ontogeny in Neanderthals and modern humans Proceedings of the Royal Society B-Biological Sciences 2007 274 1614 1125 1132 10.1098/rspb.2006.0448 PMC2189570 17311777

[B36] Klingenberg CP Heterochrony and allometry: the analysis of evolutionary change in ontogeny Biological Reviews 1998 73 1 79 123 10.1017/S000632319800512X 9569772

[B37] Webster M Zelditch ML Evolutionary modifications of ontogeny: heterochrony and beyond Paleobiology 2005 31 3 354 372 10.1666/0094-8373(2005)031[0354:EMOOHA]2.0.CO;2

[B38] Liem KF Hanken J, Hall BK Ecomorphology of the teleostean skull The skull: functional and evolutionary mechanisms 1993 Chicago: The University of Chicago Press 422 452 vol. 3

[B39] Frédérich B Lehanse O Vandewalle P Lepoint G Trophic niche width, shift, and specialization of *Dascyllus aruanus *in Toliara lagoon, Madagascar Copeia 2010 2 218 226

[B40] Froukh T Kochzius M Species boundaries and evolutionary lineages in the blue green damselfishes *Chromis viridis *and *Chromis atripectoralis *(Pomacentridae) Journal of Fish Biology 2008 72 2 451 457 10.1111/j.1095-8649.2007.01746.x

[B41] Dufour V Galzin R Colonization patterns of reef fish larvae to the lagoon at Moorea Island, French Polynesia Marine Ecology-Progress Series 1993 102 1-2 143 152

[B42] Taylor WR VanDyke GC Revised procedure for staining and clearing small fishes and other vertebrates for bone and cartilage study Cybium 1985 9 107 121

[B43] Adams DC Rohlf FJ Slice DE Geometric morphometrics: ten years of progress following the 'revolution' Italian Journal of Zoology 2004 71 1 5 16 10.1080/11250000409356545

[B44] Bookstein F Morphometric tools for landmark data: geometry and biology 1991 Cambridge University Press

[B45] Bookstein F Marcus LF, Corti M, Loy A, Naylor G, Slice D Combining the tools of geometric morphometrics Advances in morphometrics 1996 New York: Plenum Press 131 151

[B46] Rohlf FJ Marcus LF A revolution in morphometrics Trends in Ecology & Evolution 1993 8 4 129 132 10.1016/0169-5347(93)90024-J 21236128

[B47] Rohlf FJ Slice D Extensions of the Procrustes method for the optimal superimposition of landmarks Systematic Zoology 1990 39 1 40 59 10.2307/2992207

[B48] Zelditch ML Swiderski DL Sheets HD Fink WL Geometric morphometrics for biologists: A primer 2004 San Diego: Elsevier Academic Press

[B49] Rohlf FJ Marcus LF, Bello E Relative warps analysis and an example of its application to mosquito wings Contributions to morphometrics 1993 A. G-V. Madrid: Monografias del Museo Nacional de Ciencias Naturales, CSIC 131 159

[B50] Frédérich B Adriaens D Vandewalle P Ontogenetic shape changes in Pomacentridae (Teleostei, Perciformes) and their relationships with feeding strategies: a geometric morphometric approach Biological Journal of the Linnean Society 2008 95 1 92 105

[B51] Mitteroecker P Gunz P Bookstein FL Heterochrony and geometric morphometrics: a comparison of cranial growth in Pan paniscus versus Pan troglodytes Evolution & Development 2005 7 3 244 258 10.1111/j.1525-142X.2005.05027.x 15876197

[B52] Monteiro LR Multivariate regression models and geometric morphometrics: The search for causal factors in the analysis of shape Systematic Biology 1999 48 1 192 199 10.1080/106351599260526 12078640

[B53] Zelditch ML Sheets HD Fink WL Spatiotemporal reorganization of growth rates in the evolution of ontogeny Evolution 2000 54 4 1363 1371 11005302 10.1111/j.0014-3820.2000.tb00568.x

[B54] Bastir M Rosas A Facial heights: Evolutionary relevance of postnatal ontogeny for facial orientation and skull morphology in humans and chimpanzees Journal of Human Evolution 2004 47 5 359 381 10.1016/j.jhevol.2004.08.009 15530353

[B55] Darlington RB Smulders TV Problems with residual analysis Animal Behaviour 2001 62 599 602 10.1006/anbe.2001.1806

[B56] Zelditch ML Lundrigan BL David Sheets H Garland T Do precocial mammals develop at a faster rate? A comparison of rates of skull development in *Sigmodon fulviventer *and *Mus musculus domesticus* Journal of Evolutionary Biology 2003 16 4 708 720 10.1046/j.1420-9101.2003.00568.x 14632234

[B57] Mezey JG Houle D Comparing G matrices: Are common principal components informative? Genetics 2003 165 1 411 425 14504246 10.1093/genetics/165.1.411 PMC1462744

[B58] Wellington GM Victor BC Planktonic larval duration of 100 species of Pacific and Atlantic damselfishes (Pomacentridae) Marine Biology 1989 101 4 557 567 10.1007/BF00541659

[B59] Cardini A Thorington RW Postnatal ontogeny of marmot (Rodentia, Sciuridae) crania: Allometric trajectories and species divergence Journal of Mammalogy 2006 87 2 201 215 10.1644/05-MAMM-A-242R1.1

[B60] Sanfelice D de Freitas TRO The ontogeny of shape disparity in three species of Otariids (Pinnipedia: Mammalia) Latin American Journal of Aquatic Mammals 2007 6 2 139 154

[B61] Lecchini D Galzin R Spatial repartition and ontogenetic shifts in habitat use by coral reef fishes (Moorea, French Polynesia) Marine Biology 2005 147 1 47 58 10.1007/s00227-004-1543-z

[B62] McCormick MI Makey LJ Post-settlement transition in coral reef fishes: overlooked complexity in niche shifts Marine Ecology-Progress Series 1997 153 247 257 10.3354/meps153247

[B63] Osse JWM Form changes in fish larvae in relation to changing demands of function Netherlands Journal of Zoology 1990 40 1-2 362 385

[B64] Parmentier E Colleye O Fine ML Frédérich B Vandewalle P Herrel A Sound production in the clownfish *Amphiprion clarkii* Science 2007 316 5827 1006 10.1126/science.1139753 17510359

[B65] Pratchett MS Gust N Goby G Klanten SO Consumption of coral propagules represents a significant trophic link between corals and reef fish Coral Reefs 2001 20 1 13 17 10.1007/s003380000113

[B66] Frédérich B Parmentier E Vandewalle P A preliminary study of development of the buccal apparatus in Pomacentridae (Teleostei, Perciformes) Animal Biology 2006 56 3 351 372

[B67] Gluckmann I Vandewalle P Morphofunctional analysis of the feeding apparatus in four Pomacentridae species: *Dascyllus aruanus*, *Chromis retrofasciata*, *Chrysiptera biocellata *and *C. unimaculata* Italian Journal of Zoology 1998 65 421 424 10.1080/11250009809386858

[B68] Alfaro ME Bolnick DI Wainwright PC Evolutionary consequences of many-to-one mapping of jaw morphology to mechanics in labrid fishes American Naturalist 2005 165 6 E140 E154 10.1086/429564 15937739

[B69] Wainwright PC Alfaro ME Bolnick DI Hulsey CD Many-to-one mapping of form to function: A general principle in organismal design? Integrative and Comparative Biology 2005 45 2 256 262 10.1093/icb/45.2.256 21676769

[B70] Collar DC Wainwright PC Discordance between morphological and mechanical diversity in the feeding mechanism of centrarchid fishes Evolution 2006 60 12 2575 2584 17263118

[B71] Hulsey CD Wainwright PC Projecting mechanics into morphospace: disparity in the feeding system of labrid fishes Proceedings of the Royal Society of London Series B-Biological Sciences 2002 269 1488 317 326 10.1098/rspb.2001.1874 PMC1690891 11839201

[B72] Coughlin DJ Strickler JR Zooplankton capture by a coral-reef fish - an adaptive response to evasive prey Environmental Biology of Fishes 1990 29 1 35 42 10.1007/BF00000566

[B73] Kavanagh KD Alford RA Sensory and skeletal development and growth in relation to the duration of the embryonic and larval stages in damselfishes (Pomacentridae) Biological Journal of the Linnean Society 2003 80 2 187 206 10.1046/j.1095-8312.2003.00229.x

[B74] Lo-Yat A Variabilité temporelle de la colonisation par les larves de poissons de l'atoll de Rangiroa (Tuamotu, Polynésie Française) et utilisation de l'outil "otolithe" de ces larves 2002 Tahiti: Université de Polynésie française

