# Supplementary material for: Bipartite life cycle of coral reef fishes promotes increasing shape disparity of the head skeleton during ontogeny: an example from damselfishes (Pomacentridae)
Source: BMC Evol Biol. 2011 Mar 30;11:82. doi: 10.1186/1471-2148-11-82 (PMC3078888; doi:10.1186/1471-2148-11-82)
Supplement: Additional File 2 — Plots of Procrustes Distance (PD) between each specimen and the average shape of larvae on log-transformed centroid size (ln-CS). Example of Abudefduf sexfasciatus, Dascyllus aruanus and Pomacentrus pavo for each studied skeletal unit: the neurocranium, the unit «suspensorium and opercle», the mandible and the premaxilla. [file 1471-2148-11-82-S2.PPT]

## Slide 1
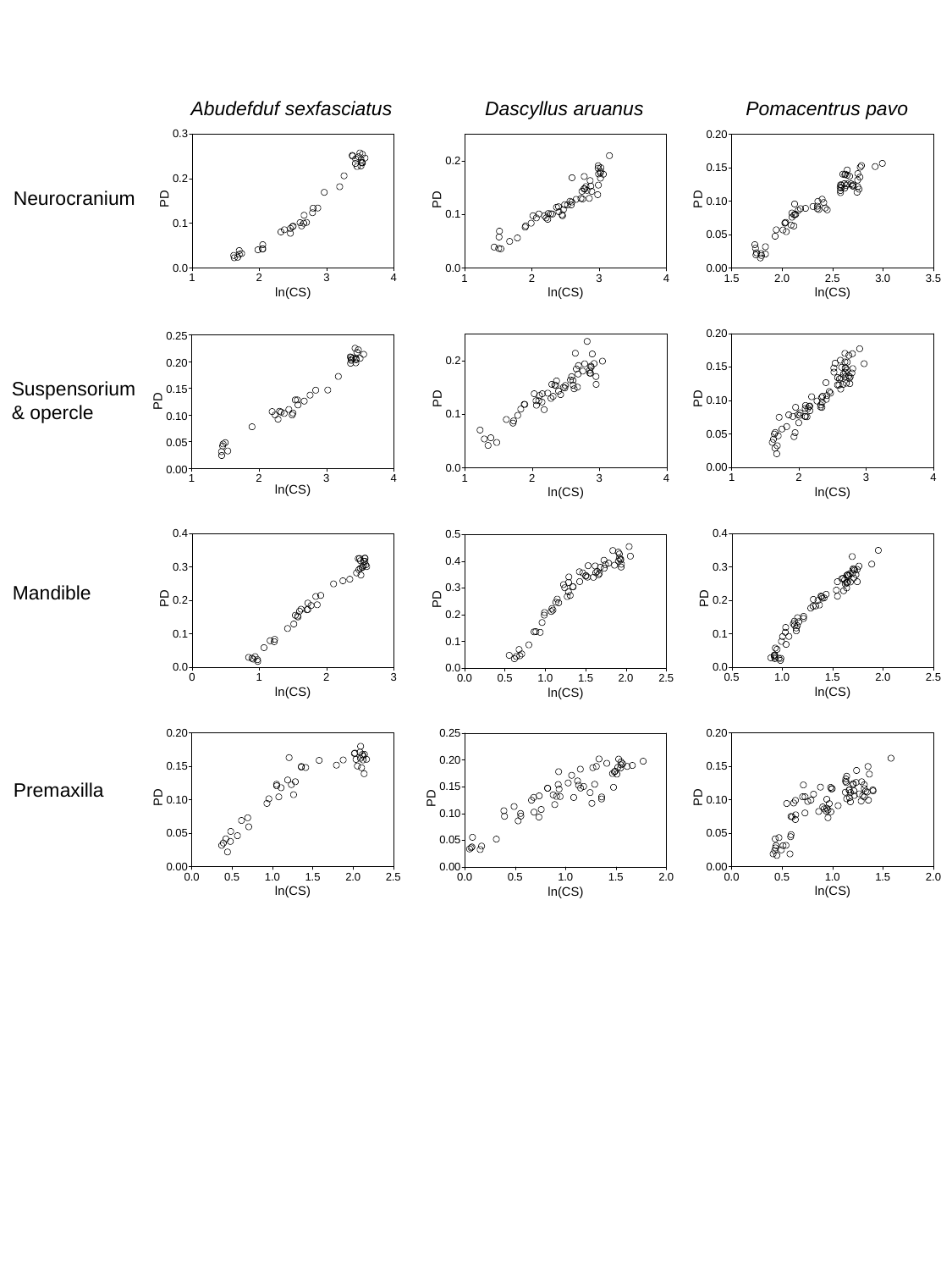

Abudefduf sexfasciatus
Pomacentrus pavo
Dascyllus aruanus
Neurocranium
Suspensorium & opercle
Mandible
Premaxilla
