# Supplementary material for: Bipartite life cycle of coral reef fishes promotes increasing shape disparity of the head skeleton during ontogeny: an example from damselfishes (Pomacentridae)
Source: BMC Evol Biol. 2011 Mar 30;11:82. doi: 10.1186/1471-2148-11-82 (PMC3078888; doi:10.1186/1471-2148-11-82)
Supplement: Additional File 3 — Illustrations of ontogenetic shape changes. TPS deformation grids illustrating the ontogenetic shape changes for each skeletal unit in every studied species [file 1471-2148-11-82-S3.PPT]

## Slide 1
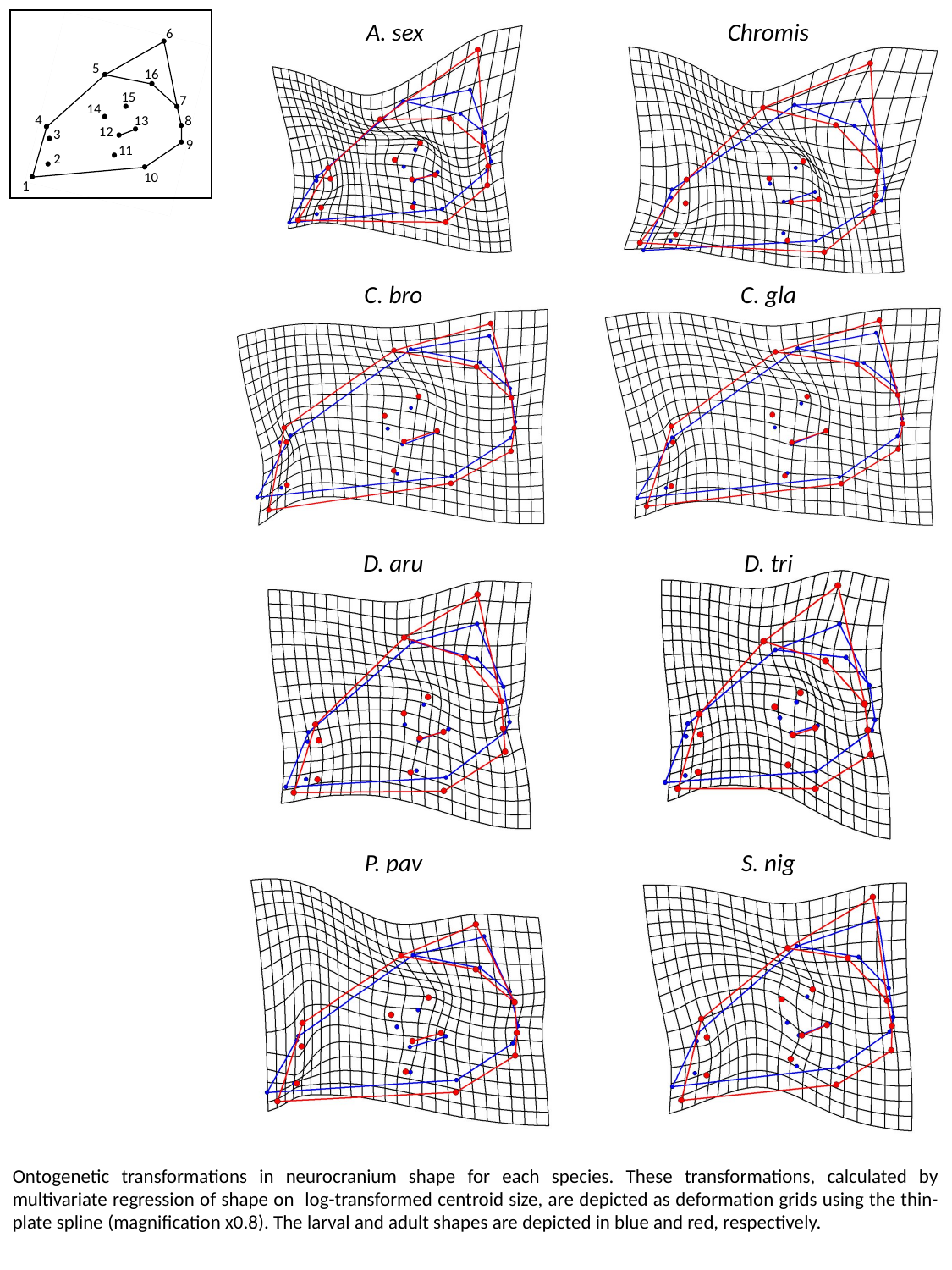

A. sex
Chromis
6
5
16
15
7
14
4
8
13
12
3
9
11
2
10
1
C. bro
C. gla
D. aru
D. tri
P. pav
S. nig
Ontogenetic transformations in neurocranium shape for each species. These transformations, calculated by multivariate regression of shape on log-transformed centroid size, are depicted as deformation grids using the thin-plate spline (magnification x0.8). The larval and adult shapes are depicted in blue and red, respectively.

## Slide 2
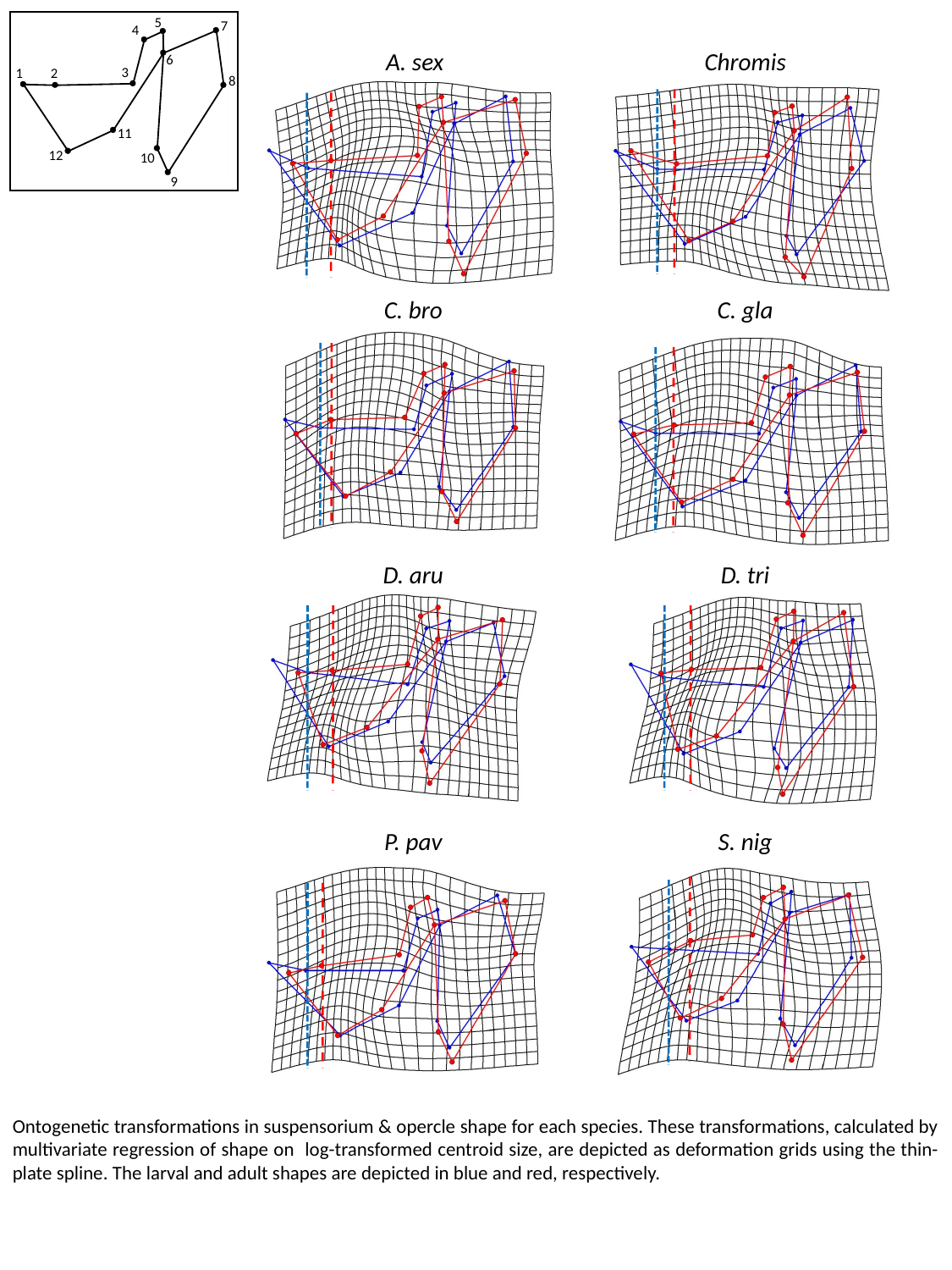

5
7
4
A. sex
Chromis
6
3
1
2
8
11
12
10
9
C. bro
C. gla
D. aru
D. tri
P. pav
S. nig
Ontogenetic transformations in suspensorium & opercle shape for each species. These transformations, calculated by multivariate regression of shape on log-transformed centroid size, are depicted as deformation grids using the thin-plate spline. The larval and adult shapes are depicted in blue and red, respectively.

## Slide 3
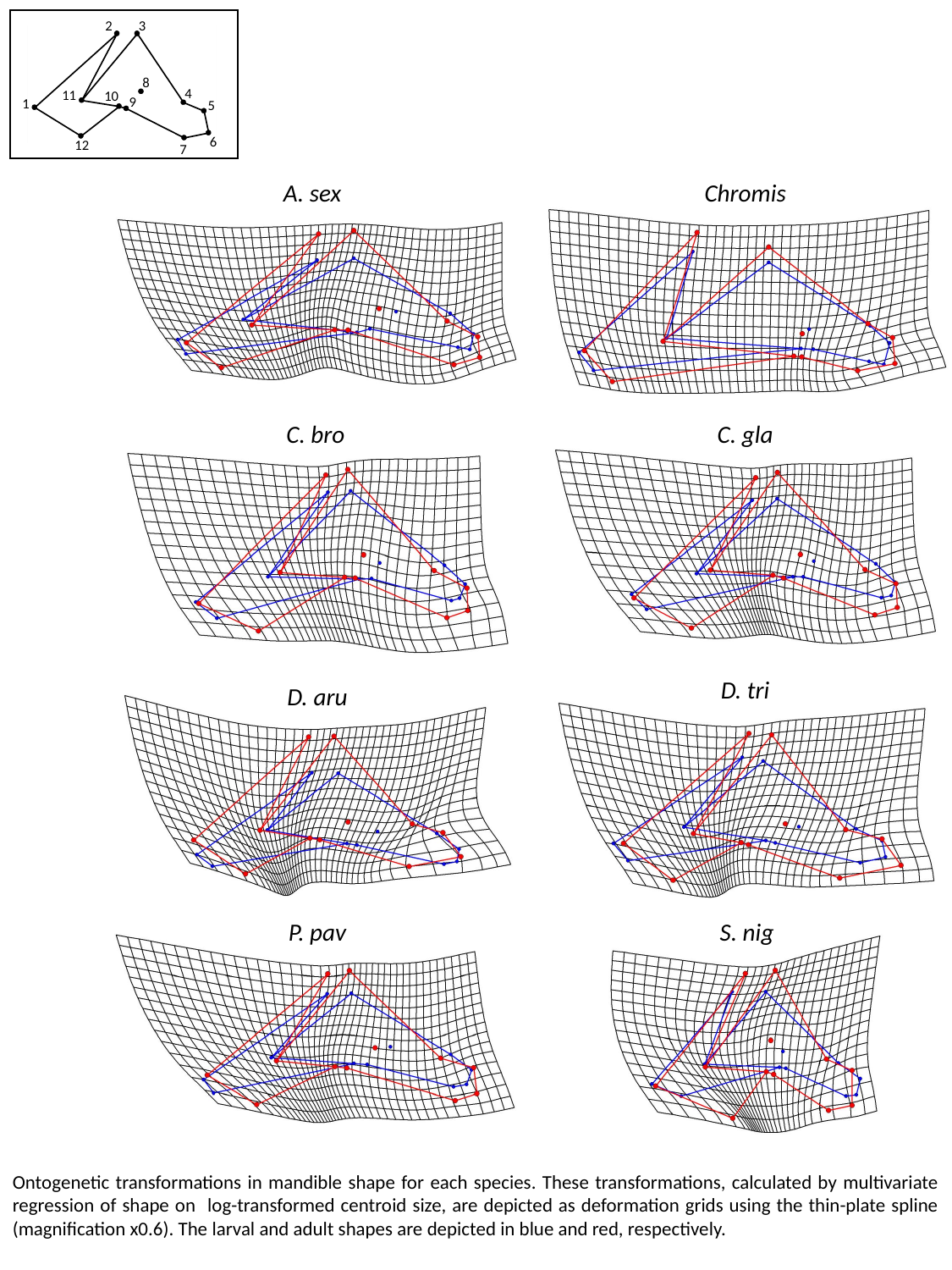

2
3
8
4
11
10
9
1
5
6
12
7
A. sex
Chromis
C. bro
C. gla
D. tri
D. aru
P. pav
S. nig
Ontogenetic transformations in mandible shape for each species. These transformations, calculated by multivariate regression of shape on log-transformed centroid size, are depicted as deformation grids using the thin-plate spline (magnification x0.6). The larval and adult shapes are depicted in blue and red, respectively.

## Slide 4
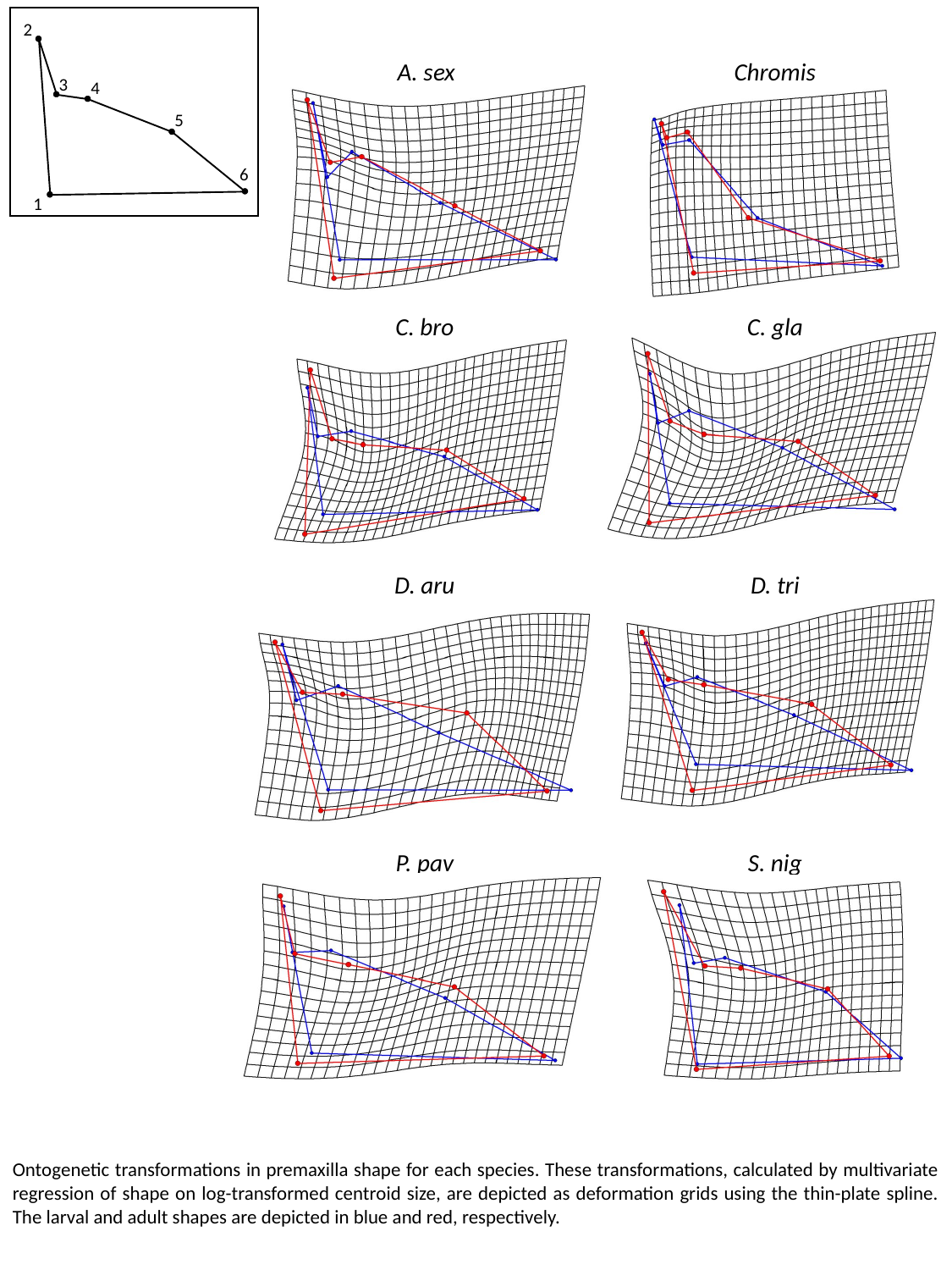

2
A. sex
Chromis
3
4
5
6
1
C. bro
C. gla
D. aru
D. tri
P. pav
S. nig
Ontogenetic transformations in premaxilla shape for each species. These transformations, calculated by multivariate regression of shape on log-transformed centroid size, are depicted as deformation grids using the thin-plate spline. The larval and adult shapes are depicted in blue and red, respectively.
